# Chromatin Landscape Is Associated With Sex-Biased Expression and Drosophila-Like Dosage Compensation of the Z Chromosome in *Artemia franciscana*

**DOI:** 10.1093/molbev/msaf085

**Published:** 2025-04-09

**Authors:** Vincent Kiplangat Bett, Minerva Susana Trejo-Arellano, Beatriz Vicoso

**Affiliations:** Institute of Science and Technology Austria (ISTA), Klosterneuburg 3400, Austria; Institute of Science and Technology Austria (ISTA), Klosterneuburg 3400, Austria; Institute of Science and Technology Austria (ISTA), Klosterneuburg 3400, Austria

**Keywords:** chromatin, dosage compensation mechanism, *Artemia*, epigenetics, sex-biased regulation, sex chromosomes

## Abstract

The males and females of the brine shrimp *Artemia franciscana* are highly dimorphic, and this dimorphism is associated with substantial sex-biased gene expression in heads and gonads. How these sex-specific patterns of expression are regulated at the molecular level is unknown. *A. franciscana* also has differentiated ZW sex chromosomes, with complete dosage compensation, but the molecular mechanism through which compensation is achieved is unknown. Here, we conducted CUT&TAG assays targeting 7 post-translational histone modifications (H3K27me3, H3K9me2, H3K9me3, H3K36me3, H3K27ac, H3K4me3, and H4K16ac) in heads and gonads of *A. franciscana*, allowing us to divide the genome into 12 chromatin states. We further defined functional chromatin signatures for all genes, which were correlated with transcript level abundances. Differences in the occupancy of the profiled epigenetic marks between sexes were associated with differential gene expression between males and females. Finally, we found a significant enrichment of the permissive H4K16ac histone mark in the Z-specific region in both tissues of females but not males, supporting the role of this histone mark in mediating dosage compensation of the Z chromosome.

## Introduction

The males and females of many species differ in their morphology, physiology, and/or behavior. While the primary signal for sex determination is encoded on the sex chromosomes in species with genetic sex determination, sexual differentiation is thought to rely on differential gene expression (sex-biased genes) between males and females throughout the genome (Parsch and Ellegren 2013). The nature and extent of sex-biased gene expression are quite diverse across taxa and can vary with tissues and developmental stages (Grath and Parsch 2016), and understanding how such dimorphism in expression evolves and is regulated has been a longstanding goal. Some dimorphic genes and traits are directly regulated by the sex-determination cascade. For instance, in *Drosophila*, the acquisition of sexually dimorphic foreleg sex combs is associated with the expression of the sex-determining transcription factor *dsx* (Barmina et al. 2005). Similarly, within the *Drosophila* brain, specific neurons exhibit sex-specific isoforms of the transcription factor *Fru*, a pivotal regulator of sexual dimorphism responsible for directing male-specific courtship behaviors (Dickson 2008). However, the majority of sex-biased genes are not directly bound by members of the sex-determination pathway, and more complex mechanisms must be at play. The configuration of the chromatin structure, which can be open and associated with transcription, or closed and associated with silencing, and can be shaped by the sex-determination pathway (Tachibana 2016), is another regulatory layer that is becoming increasingly appreciated as a source of dimorphism.

In the brown algae *Ectocarpus,* there is a correlation between differential chromatin signatures and sex-biased genes crucial for sexual differentiation (Gueno et al. 2022). Moreover, in mammalian fetal germ cells, the sex-specific chromatin configuration corresponds to anticipated differential gene expression and morphological differences between the sexes during later stages of development (Lesch and Page 2013). Finally, recent research on 2 closely related *Drosophila* species highlighted the involvement of the open chromatin mark H3K4me3 and closed chromatin mark H3K27me2me3 in maintaining sex-biased gene expression (Nanni et al. 2023). In particular, this work supported the model of “Open in Same sex and/or Closed in Opposite” for sex bias (Nanni et al. 2023), the idea that a gene will be in open chromatin only in the sex where it is primarily expressed (Brown and Bachtrog 2014). However, only 3 histone marks were sampled, and only the head, an organ with limited sexual dimorphism, was considered, such that the relevance of this model to broader patterns of molecular dimorphism is still unclear.

Differences in the male and female chromatin landscape are also crucial to maintain gene expression balance in species with differentiated sex chromosomes, such as the X and Y of mammals. Sex chromosomes originally evolve from ordinary pairs of autosomes after they acquire sex-determining gene(s), which is often followed by recombination suppression over part of the chromosome and degeneration of the Y (in XY system) or W (in ZW systems, where females are ZW while male are ZZ) (D. Charlesworth, Charlesworth, and Marais 2005). The degeneration of the Y (or W) results in different copy numbers of X/Z-linked and autosomal genes in the heterogametic sex, which can cause imbalances in gene expression between sex chromosomes and autosomes (Grath and Parsch 2016). This is thought to be deleterious, as many gene networks and protein complexes involve both X-linked and autosomal gene products (B. Charlesworth 1978; Veitia 2005). Dosage compensation mechanisms have consequently evolved independently in many eukaryotic taxa. The process is thought to begin with the restoration of ancestral expression through increased gene expression on the X (or Z) chromosome in the heterogametic sex. When this increase is not sex-specific, it causes the overexpression of X/Z-linked genes in the homogametic sex, triggering the secondary evolution of mechanisms to suppress X (or Z) expression and restore balance between the sex chromosome and autosomes in the homogametic sex (and consequently between the sexes) (Lucchesi, Kelly, and Panning 2005). Such mechanisms may affect the entire sex chromosome (chromosome-wide dosage compensation) or only a subset of dosage-sensitive genes (gene-by-gene compensation) (Gu and Walters 2017).

Chromosome-wide dosage compensation can therefore be achieved by upregulating X-linked gene expression in the heterogametic sex (e.g. in *Drosophila*) or reducing transcription in the homogametic sex (Muyle et al. 2021). In placental mammals, one of the entire X chromosomes is randomly inactivated in females, while concurrently upregulating a subset of X-linked genes in both sexes (Lucchesi, Kelly, and Panning 2005; O’Neill et al. 2008). In *Caenorhabditis elegans,* downregulation is achieved by a two-fold reduction in transcription rates from both X chromosomes of the homogametic sex (Lau and Csankovszki 2015). Although these compensation mechanisms vary by species, they all lead to the equalization of expression between the sexes, and they are usually accompanied by changes in the sex-specific chromatin structure (Lucchesi and Kuroda 2015; Marin et al. 2017). Studies on dosage compensation have historically been concentrated on species with male heterogamety, which typically show chromosome-wide dosage compensation. On the other hand, ZW systems often have partial dosage compensation mechanisms, where only dosage-sensitive genes are compensated (Itoh et al. 2007; Vicoso et al. 2013). Lepidopteran insects are an important exception to this pattern, as they have a nematode-like dosage compensation that affects the Z chromosome globally (Gu and Walters 2017). However, a recent study on monarch butterflies with young sex chromosomes showed that the ancestral-Z and the neo-Z chromosome unexpectedly showed two distinct modes of dosage compensation mechanisms (Gu et al. 2019). The newly derived Z segment had a *Drosophila*-like dosage compensation mechanism while the transcription of ancestral-Z genes was downregulated to nearly halve the ZZ genes in males as reported in other Lepidopteran species (Gu et al. 2019). Why X/Z:autosome imbalances lead to such diverse compensation mechanisms is poorly understood, in part due to the limited number of systems that have been characterized in detail.

Here we address these questions in *Artemia franciscana*, a crustacean which exhibits extensive morphological sexual dimorphism (in particular male heads possess protective claspers for mating which are absent in females). A significant number of genes show differential expression in the heads and gonads of males and females, predominantly in gonadal tissue (Huylmans et al. 2019). To understand the molecular basis of this dimorphism, and of crustacean gene regulation in general, we characterized the chromatin landscape of this species using various active and repressive histone marks, in heads and gonads of the two sexes. Furthermore, *Artemia franciscana* has a pair of ZW sex chromosomes which have acquired complete dosage compensation (Huylmans et al. 2019; Bett et al. 2024). However, the underlying molecular mechanisms are unknown. We investigated how epigenetic modifications may mediate equalization of gene expression between sexes despite their differences in gene copy numbers.

## Results

### Correlation of Histone Modifications With Expression

We generated CUT&TAG datasets from the heads and gonads of *A. franciscana*, focusing on 7 distinct histone modifications. These post-translational histone modifications offer a comprehensive biological annotation across *A. franciscana* genome, encompassing heterochromatin, active transcription, and polycomb-mediated repression (Table 1).

**Table 1:** Histone marks profiled in this study together with their role in transcription and associated functions in the regulation of sex chromosomes

Histone mark	Active or repressive (Kimura 2013)	No. of replicates in each tissue	Known function (Bannister and Kouzarides 2011)	Regulation of sex chromosomes
H3K27ac	Active	2	Associated with active enhancers	Involved in regulation of sex-determination and differentiation genes in chicken (Jiang et al. 2022)
H3K4me3	Active	2	Marks active promoters	Enriched in the Z-specific region of Schistosome (Picard et al. 2019)
H4K16ac	Active	2	Highly enriched on TSS	Enriched on single male X of Drosophila (Gelbart et al. 2009), neo-Z female of Monarch Moth (Gu et al. 2019)
H3K36me3	Active	2	Enriched toward the 3′ of active genes	Transcriptional upregulation of the male X of Drosophila (Bell et al. 2008)
H3K27me3	Repressive	3	Transcriptional repression of developmentally regulated genes	Accumulates on the inactivated X-chromosome of females of placental mammals (Kelsey et al. 2015)
H3K9me2	Repressive	2	Marks constitutive heterochromatin	Enriched on neo-Y regions with a high repeat content in *Drosophila miranda* (Zhou et al. 2013)
H3K9me3	Repressive	2	Associated with heterochromatin, satellite repeats, gene-poor regions	Modulates heterochromatin integrity of Y chromosome (Brown, Nguyen, and Bachtrog 2020)

**Fig. 1.**
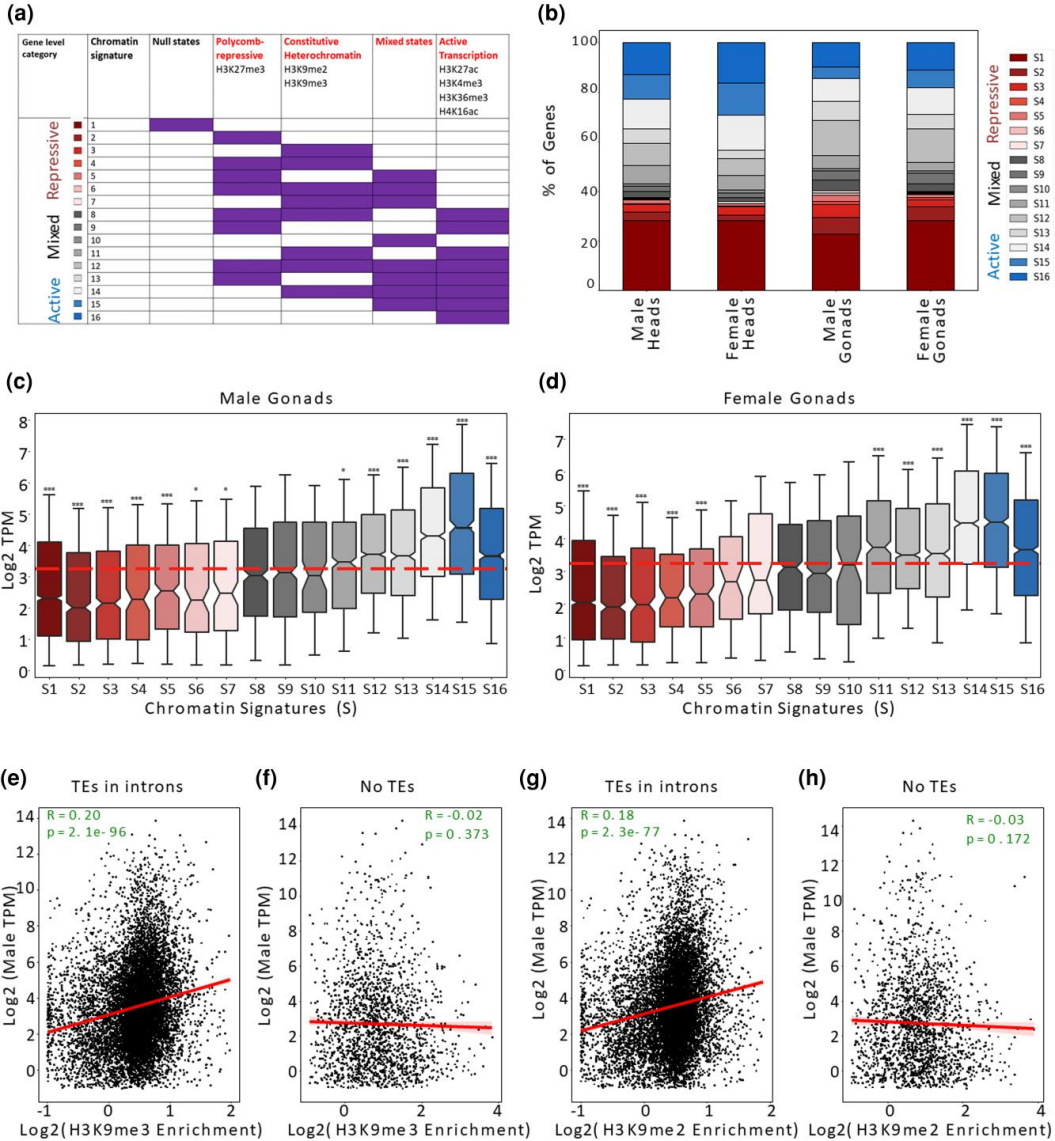
The chromatin landscape and correlation with expression in *A. franciscana*. a) Active, repressive, and mixed chromatin signatures (S1 to S16) were inferred based on the combination of emission states (supplementary fig. S2, Supplementary Material online) of genes. b) The proportion of chromatin signatures (S1 to S16) across autosomal and pseudoautosomal genes annotated in the *A. franciscana* genome in each tissue and sex. c) Expression of genes with different chromatin signatures (S1 to S16) in male gonads d) Expression patterns of genes of different chromatin signatures (S1 to S16) in female gonads. The dotted line indicates the median expression of all annotated genes. Wilcoxon signed-rank test was performed between genes of particular chromatin signature and the expression of the genes of the rest of the genome (autosomes and pseudoautosomal genes), with * denoting a *P*-value < 0.05, ** is *P*-value < 0.005, and *** is *P*-value < 0.0005. Similar plots are provided for expression in heads in supplementary Figs. 4a and 4b, Supplementary Material online. Panels E and F show the correlation of male head expression with H3K9me3 for genes with e) or without f) TEs in their introns. Panels G and H show the correlation of male head expression with H3K9me2 for genes with g) or without h) TEs in their introns. The few genes with TEs overlapping their exons were excluded from the analysis. Similar plots using female heads and gonads expression values are provided in supplementary Figs. 4C-4N, Supplementary Material online.

Using Spectacle (Song and Chen 2015), we applied a multivariate hidden Markov algorithm to infer 12 chromatin states across the *A. franciscana* genome based on the presence and absence of these 7 histone modifications (supplementary figs. S1, S2, and S3, Supplementary Material online). While these chromatin states are informative about functional elements along the genome, they are difficult to correlate directly with expression, as multiple states can be simultaneously found on each gene. Following Gueno et al. (Gueno et al. 2022), we therefore obtained gene-level chromatin signatures from the combination of states found on each gene. First, the chromatin states (E1 to E12) were clustered into 4 functional categories: (a) active transcription, marked by the enrichment of these 4 active histone modifications or their combinations (H3K36me3, H3K27ac, H3K4me3, and H4K16ac); (b) polycomb-mediated repression, identified by the enrichment of H3K27me3; (c) heterochromatin (if they were enriched for H3K9me2 and/or H3K9me3); (d) mixed chromatin, where both active and repressive chromatin marks are enriched; and we also included a background null state, characterized by the absence of distinct chromatin features. All possible combinations of functional categories of chromatin states, along with the background null state, resulted in a total of 16 gene-level chromatin signatures (Fig. 1a, S1 to S16).

These chromatin signatures can then again be broadly classified as active (if the genes contain only active chromatin states and mixed chromatin states, S15 to S16), or repressive (if genes exhibit either the background null state or a combination of repressive and mixed states, S1-S7). Genes with only mixed states, or with both active and repressive marks, were classified as having a mixed chromatin signature (S8 to S14). In order to avoid confounding effects of the sex chromosome chromatin (see section 3), we focused our analysis on genes located in the autosomal and pseudoautosomal regions. Heads were enriched for active chromatin signatures compared to gonads (Fig. 1b, Fisher's Exact, *P*-value = 4.65e-77 in male and *P*-value = 1.31e-142 in female), while repression-associated chromatin signatures were more prevalent in gonads (Fig. 1b, Fisher's exact, *P*-value = 4.81e-07 in male and *P*-value = 4.58e-46 in female).

In order to explore the functional relevance of this classification, we compared the expression of genes associated with different chromatin signatures in both somatic and gonadal tissues of male and female *A. franciscana*. Genes with repressive signatures (S1 to S7) display significantly lower expression levels (*P*-value < 0.001, Wilcoxon rank sum tests) than genes with other chromatin signatures. In contrast, genes associated with active chromatin signatures (S15 and S16, enriched for H3K36me3, H3K27ac, H3K4me3, and/or H4K16ac) show higher transcript abundance (*P*-value < 0.0001, Wilcoxon rank sum tests; Fig. 1c and 1d, supplementary figs. S4 a and S4b, Supplementary Material online).

Although both H3K27me3 and H3K9me2/me3 are typically repressive (Kimura 2013), H3K9me3 is required for the transcription of certain genes located in heterochromatic regions of the Drosophila genome (Riddle et al. 2011; Ninova, Fejes Tóth, and Aravin 2019; Ninova et al. 2020). This is thought to be driven by the need to repress disruptive transcription from promoters of transposable elements located in their intronic regions (Ninova et al. 2020). The high expression of genes in certain mixed states led us to wonder whether heterochromatin marks might behave similarly in the context of the highly repetitive genome of *A. franciscana* (Bett et al. 2024). In particular, genes in signature S11 (with chromatin marks associated with heterochromatin and any/all active histone marks) and S14 (heterochromatin + active + mixed states) have higher expression than the genome-wide median (*P*-value < 2.6e-63 and *P*-value < 2.8e-115, male and female gonads, respectively, Fig. 1c and 1d, supplementary figs. S4a and S4b, Supplementary Material online), suggesting a relatively permissive role of constitutive heterochromatin in *Artemia*. To investigate if this is related to the presence of transposable element sequences in intronic regions, we separated genes into those carrying at least one transposable element in their introns (“TE in introns”), and those not overlapping with any transposable elements (“No TE”). We then correlated the expression of these two categories of genes with H3K9me3 and H3K9me2 enrichment (Fig. 1 e-f, Fig. 1 g-h, respectively). “TE in introns” genes showed a positive correlation for both histone marks (*P*-value < 8.7e-55 and *P*-value < 2.3e-77, respectively, Spearman correlation), while “No TE” did not. This difference in the correlation coefficients of H3K9me2/3 enrichment and gene expression was significantly different between TE-carrying genes and genes without transposable elements (TEs) for both tissues and sexes (*P*-value < e-10 to e-30 in all cases, Fisher r-to-z transformation of the correlation coefficients, supplementary table S1, Supplementary Material online).

### Chromatin Landscape and Sex-biased Gene Expression

**Fig. 2.**
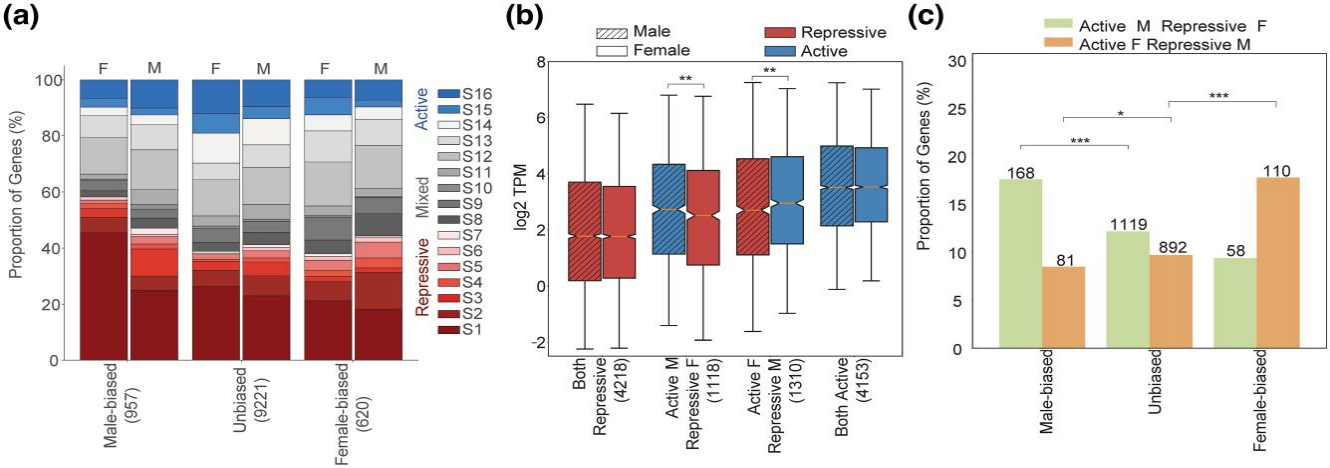
Sex-biased expression is associated with differential chromatin states in males and females. a) Composition of different categories of sex-biased gene expression in different chromatin signatures in gonads (adjusted *P*-value < 0.05, foldchange >2, and TPM >0.1). b) Expression levels of genes with active-associated or repressive-associated chromatin signatures in both males and females or contrasting chromatin states between the sexes. Statistical significance was evaluated using a pairwise Wilcoxon rank sum test with * denoting a *P*-value < 0.05, ** as *P*-value < 0.005, and *** as *P*-value < 0.0005. c) Proportion of genes with contrasting chromatin states in males and females, for different categories of sex-biased expression in gonads of *A. franciscana* (adjusted *P*-value < 0.05, foldchange >2, and TPM >0.1). Statistical significance was evaluated using a pairwise Wilcoxon rank sum test with * denoting a *P*-value < 0.05, ** as *P*-value < 0.005, and *** as *P*-value < 0.0005.

We examined how chromatin influences sex-specific gene expression patterns by analyzing two replicated RNA samples from the heads and gonads of both sexes in *A. franciscana*. We again focused only on (pseudo)autosomal genes. Only 10 genes exhibited female bias and 10 genes male bias in heads (adjusted *P*-value < 0.05, fold change > 2, TPM > 0.1; supplementary table S2, Supplementary Material online). No further analyses of sex bias were performed in this tissue, as these small numbers do not allow for meaningful comparisons (but qualitative patterns are similar to gonads, supplementary table S2, Supplementary Material online, supplementary fig. S5, Supplementary Material online). Gonads, on the other hand, displayed 979 male-biased and 633 female-biased genes (adjusted *P*-value < 0.05, fold change > 2, TPM > 0.1; supplementary table S2, Supplementary Material online). Male-biased genes were more often associated with repressive chromatin signatures (S1 to S7) in ovaries (59%) than in testes (47%, *P*-value < 7.52e-07, Fisher's exact test, Fig. 2a), while the opposite was true for female-biased genes (44% in testis vs. 36% in ovaries, *P*-value < 0.02, Fisher's Exact test, Fig. 2a).

To directly test whether differences between the male and female chromatin state of each gene influenced their sex-specific pattern of expression, genes were classified based on the active or repressive status of their chromatin signature in the two sexes. In order to be able to classify all sex-biased genes, and because some H3K9me2/3-associated chromatin states were effectively active rather than mixed (see previous section), we classified all chromatin signatures whose associated genes had expression significantly above the genomic median as active (S11 to S16), whereas all others were classified as repressive (Fig. 2b). The “open in same sex/closed in opposite” model (Nanni et al. 2023) predicts that many male-biased genes should show active chromatin signatures in males and repressive signatures in females (and conversely for female-biased genes). This was indeed the case, with female-biased genes for female-active/male-inactive states (*P*-value < 0.0001 in gonads, chi-square test, Fig. 2c, supplementary figs. S6a-S6b, Supplementary Material online) and male-biased genes being enriched for male-active/female-inactive states (*P*-value < 6.7e-06 in gonads, chi-square test, Fig. 2c, supplementary figs. S6c and S6d, Supplementary Material online). Gene Ontology (GO) enrichment terms of genes that were both female-biased and male-biased with contrasting chromatin-associated signatures between sexes in gonads were enriched in functions related to regulation of gene expression, cellular processes, or system development (supplementary table S3, Supplementary Material online in female, supplementary table S4, Supplementary Material online in males).

### Epigenetics of Dosage Compensation in *Artemia franciscana*

**Fig. 3.**
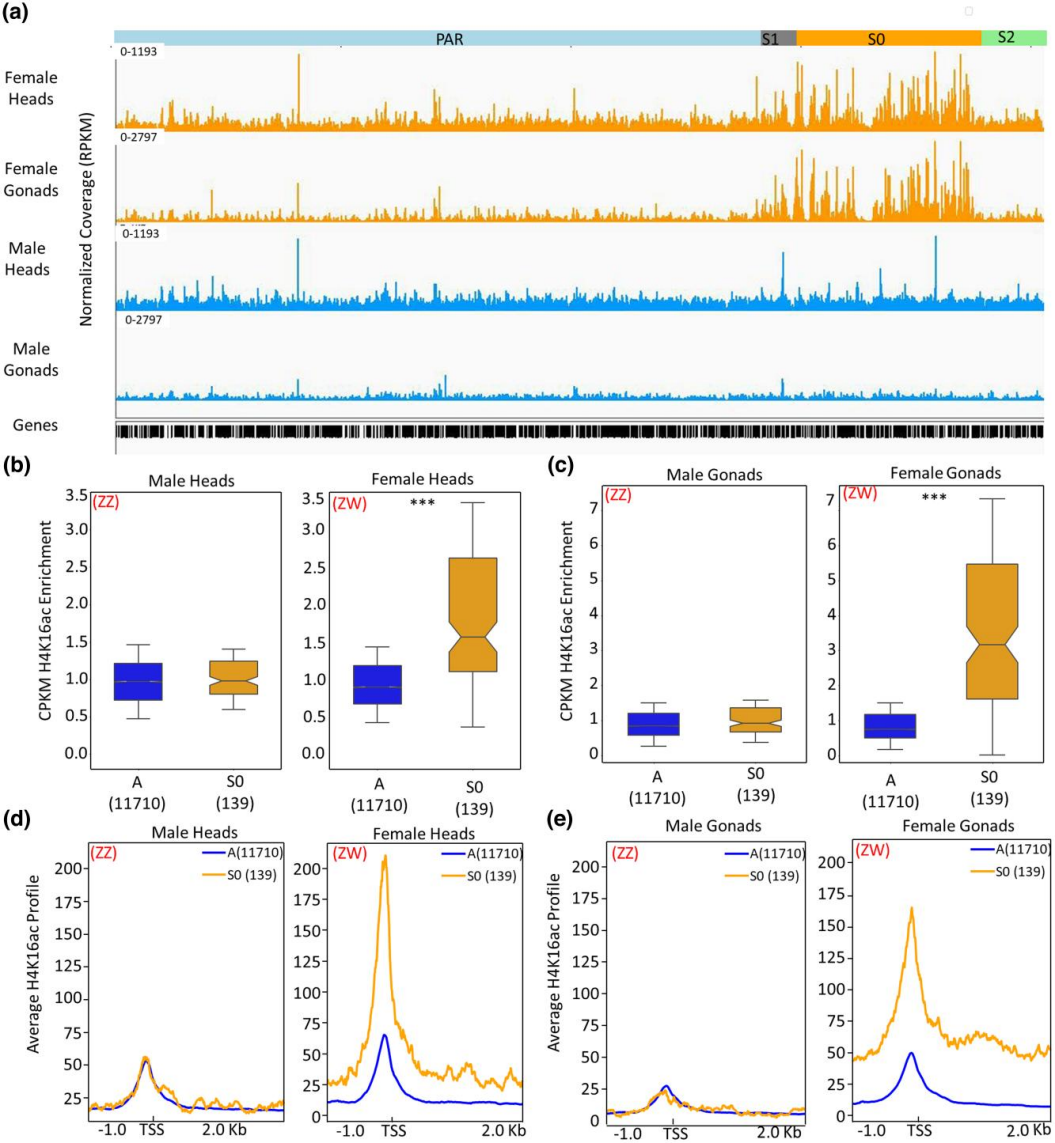
H4K16ac underlies dosage compensation of the Z. a) Schematic regions of the Z chromosome and genome browser view of H4K16ac in heads and gonads of both males and females. b and c) Boxplots of Normalized H4K16ac enrichment across genes in Z-specific (S0) and autosomes in head b) and gonadal tissue (c). Statistical significance was assessed using a pairwise Wilcoxon rank sum test with * denoting a *P*-value < 0.05, ** as *P*-value < 0.005 and *** as *P*-value < 0.0005. d and e) Average profile enrichment of H4K16ac around TSS (1 kb upstream and 2 kb downstream of TSS) of genes in the Z-specific region (S0) and autosomes of head d) and gonad e) tissues in both males and females.

*Artemia franciscana* possesses a pair of ZW sex chromosomes, where a well-differentiated region of the Z chromosome (the “S0” region, Fig. 3a) has evolved complete dosage compensation (Huylmans et al. 2019; Bett et al. 2024). In order to determine how chromatin structural changes may regulate dosage compensation in this species, we first assessed the enrichment patterns of normalized histone marks for both replicates across each gene, comparing Z-specific genes (located in the S0 region of the Z chromosome, 139 genes, TPM > 0.5) with autosomal genes in each sex and tissue (supplementary figs. S7 and S8, Supplementary Material online). The normalized signal distribution of most active histone marks (H3K36me3, H3K27ac, and H3K4me3) and repressive histone marks (H3K9me2, H3K9me3, and H3K27me3) were not statistically significantly different between autosomes and the Z-specific region in gonads in both males and females (*P-*value > 0.05, Wilcoxon rank sum tests, supplementary figs. S7 and S8, Supplementary Material online). In heads, only histone marks H3K27ac and H3K9me3 were statistically depleted on the Z-specific region of females compared to the autosomes (*P*-value = 0.028 and *P-*value = 0.002, respectively). However, individual assessments of the two replicates in this female somatic tissue of these histone marks (H3K27ac and H3K9me3) gave inconsistent differences between the autosomes and the Z-specific region, with one of the replicates being significant while the other is insignificant (*P-*value = 0.58 and *P-*value = 1, Wilcoxon rank sum test in H3K27ac and H3K9me3, respectively, supplementary fig. S9, Supplementary Material online), suggesting these may be an artifact rather than true biological signals.

On the other hand, the active histone mark H4K16ac was enriched approximately two-fold on Z-specific genes (S0) relative to autosomal genes in the heads, and more than two-fold in the gonads in ZW females (*P-*value < 2.2e-24, Wilcoxon rank sum tests, Fig. 3b, Fig. 3c, supplementary fig. S9, Supplementary Material online). By contrast, the profile of this histone mark was similar between genes in Z-specific and autosomal regions of ZZ males (*P-*value = 1, Wilcoxon rank sum tests, Fig. 3b, Fig. 3c, supplementary fig. S9, Supplementary Material online). To explore how the active chromatin mark H4K16ac is distributed along the length of gene bodies, we examined its average enrichment patterns, spanning 1 kb upstream to 2 kb downstream of the transcription start site (TSS) for genes on both the Z-specific region (S0) and on the autosomes. We used normalized coverage values from replicates from each tissue in each sex. We found that the enrichment is present not only at the TSS but across the entire length of the gene body (Fig. 3d, Fig. 3e). This enrichment of H4K16ac in female relative to male tissues was exclusive to the region (S0) of the Z chromosome (Fig. 3a, supplementary figs. S10 and S11, Supplementary Material online). It should be noted that the role of H4K16ac in *Artemia* dosage compensation has been independently inferred by the Keller-Valsecchi lab (Zimmer et al. 2025).

**Fig. 4.**
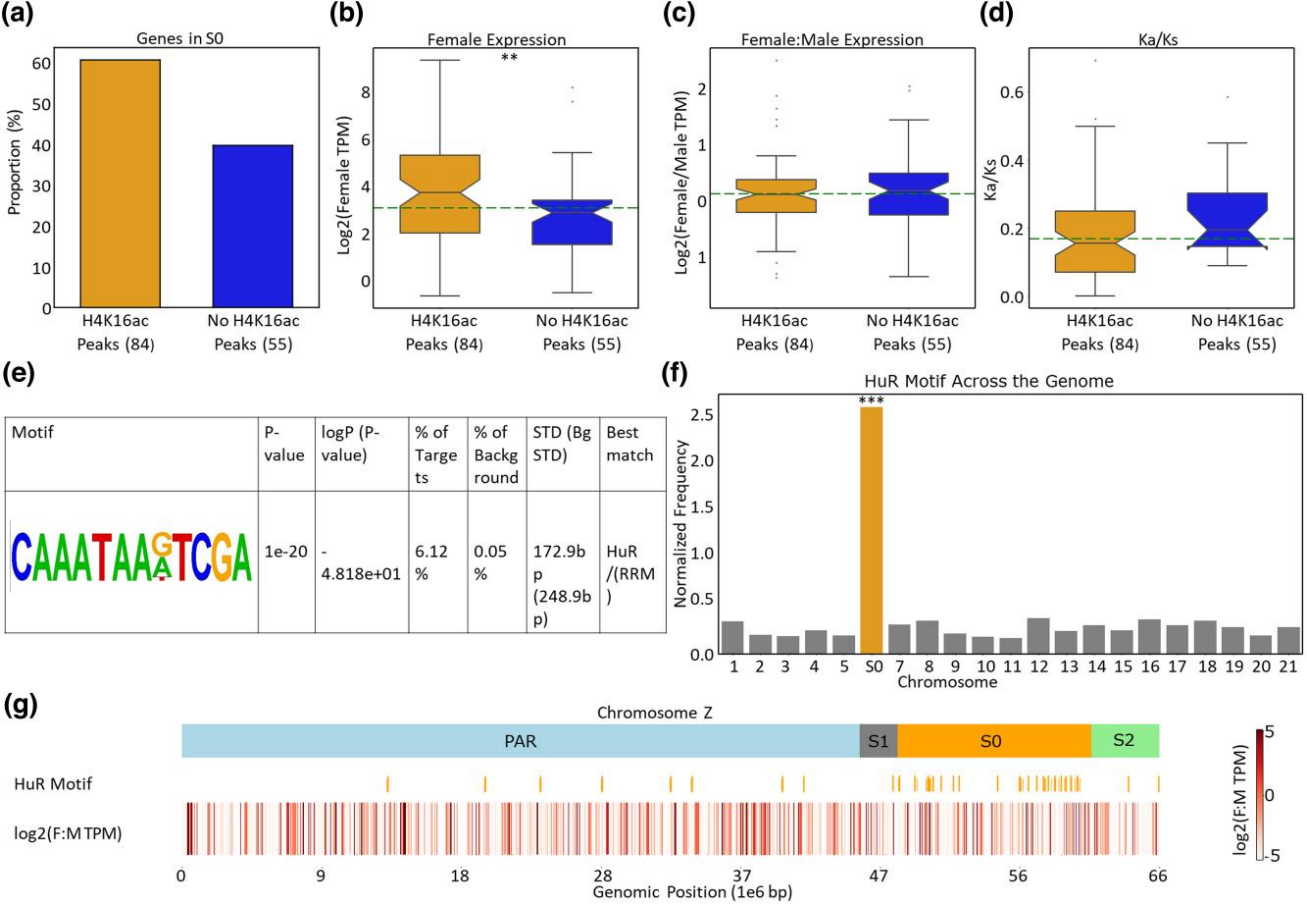
H4K16ac peaks in the S0 region and their association with functional genomic features. a) Proportion of genes in the Z-specific region (S0) that either intersect or do not intersect with H4K16ac peaks in female heads. b) Expression of genes in the Z-specific region (S0) with and without H4K16ac peaks in female heads. The significance of the Wilcoxon rank sum test ** is *P*-value < 0.005. c) Log2 of female:male expression ratio of S0 genes with and without H4K16ac peaks in heads. d) The distribution of Ka/Ks of S0 genes with and without overlap with H4K16ac peaks. e) Motif with the most significant enrichment in S0 H4K16ac peaks relative to the rest of the genome. f) Frequency (in number per million base pairs) of the motif described in e) on different chromosomes. *** denotes *P* < 0.005, obtained by resampling *N* chromosomal loci randomly 10,000 times, where *N* is the number of peaks found throughout the genome. g) Schematic regions of the Z chromosome, HuR motif distribution patterns, and their relationship to female-to-male expression ratios in head tissue. Similar plots for gonads are provided in supplementary fig. S12, Supplementary Material online.

These patterns may reflect a global enrichment in H4K16ac throughout the S0, or the targeting of a subset of genes by the dosage compensation machinery. To obtain further insights into the architecture of dosage compensation of this group, we first called H4K16ac peaks and found that over 50% of expressed S0 genes overlapped with an H4K16ac peak (84 out of 139 genes, Fig. 4a). Genes overlapping H4K16ac peaks showed increased transcript abundance compared to those outside of peaks (*P-*value = 0.002, Wilcoxon rank sum tests, Fig. 4b), and a trend toward higher sequence conservation (inferred from the nonsynonymous to synonymous divergence ratio, Ka/Ks; the difference was however not significant (Fig. 4d)). However, the expression ratio between females and males did not differ between genes with and without H4K16ac peaks (*P-*value = 0.709, Wilcoxon rank sum tests, Fig. 4c), suggesting that balancing of expression occurs globally for the region.

In order to investigate how H4K16ac may be regulated, we conducted a DNA-binding motif enrichment analysis on the set of H4K16ac peaks (333 and 294 peaks in heads and gonads, respectively) that were intersecting with S0 genes expressed in female tissues (TPM > 0.5 in either female gonads or heads). The HOMER analysis revealed many sequence motifs that were significantly enriched (*P*-value <1e-10) in sequences containing H4K16ac enriched peaks in the Z-specific region (S0) (Fig. 4e, supplementary tables S5 and S6, Supplementary Material online). The most significantly enriched motif in both head and gonad peaks was an AT-rich motif previously identified as a binding motif for Human antigen R (HuR(RRM)) (*P*-value = 1e-19). This motif comprises over 6% of peak regions, but less than 1% of the rest of the genome. Importantly, it is also highly enriched in the S0 region compared to other chromosomes (*P*-value < 0.0001, resampling with 10000 bootstraps, Fig. 4f) and to the rest of the Z chromosome (Fig. 4g), unlike the other 2 most enriched motifs in the peaks. Taken together, these results suggest that a dosage compensation mechanism likely targets the S0 region at specific binding motifs, where it promotes H4K16ac, and global upregulation of gene expression in the region, mirroring the well-known mechanism of Drosophila.

## Discussion

Here, we present the first comprehensive analysis of the chromatin landscape of a crustacean species with genetic sex-determination systems. In agreement with the idea that the chromatin landscape may play a regulatory role in gene expression, repressive-associated chromatin signatures were consistently associated with lower transcript abundance compared to active-associated chromatin signatures. Additionally, distinct chromatin states between males and females were associated with sex-biased gene expression in both somatic and gonadal tissues. Finally, we provide evidence that changes in chromatin landscape are likely used in *A. franciscana* to equalize the expression of genes between Z-specific and autosomes and between sexes.

### Expression of Gene Regulation in a Highly Repetitive Genome

We sampled a broad range of known active and repressive histone marks in order to get a full overview of a crustacean chromatin landscape. We combined these with published RNA-sequencing data (Huylmans et al. 2019) to characterize epigenetic regulation in this clade. The hatching and growth conditions were slightly different in the 2 studies (in particular, the salinity was 30 to 60 g/l before, and 50 g/l here), but most histone marks yielded the expected associations with expression (supplementary figs. S13 to S17, Supplementary Material online), suggesting this did not strongly impact the conclusions. Interestingly, both repressive marks H3K9me2 and H3K9me3 were overall enriched on genes with high expression (supplementary figs. S18 and S19, Supplementary Material online). Our combinatorial analysis suggests that these two marks of heterochromatin may have distinct associations with transcription when they act alone (repressive role) or in combination with chromatin marks associated with active transcription (permissive role). This is in line with Feng et al. (Feng et al. 2020) who reported that the genes exhibiting the acquisition of H3K9me3 became heterochromatic, but required the concurrent depletion of H3K4me3 and H3K27ac to undergo transcriptional repression. This combinatorial mode of action may be particularly important in *A. franciscana,* since nearly two-thirds of its genome consist of repetitive elements that need to be condensed while allowing for gene transcription to occur (Bett et al. 2024). Actively transcribed genes situated within constitutive heterochromatin in *Drosophila melanogaster* necessitate this chromatin environment for their appropriate expression, as their activity is compromised upon relocation to open chromatin regions (Ninova, Fejes Tóth, and Aravin 2019). Moreover, the depletion of H3K9me3 leads to the transcriptional suppression of a considerable amount of genes housed within constitutive heterochromatin (Ninova, Fejes Tóth, and Aravin 2019). This may be due to the presence of transposable elements in the introns of these genes, whose promoters need to be repressed through H3K9me3 deposition to avoid spurious intronic transcription (and consequent downregulation of expression of the gene) (Ninova et al. 2020). The presence of TEs within introns in *Artemia franciscana* may play a similarly crucial role in driving the positive correlation between H3K9me2/me3 enrichment and gene expression, supporting the idea that heterochromatization may allow for proper gene regulation in highly repetitive regions. Interestingly, in yeast, H3K9me2 and H3K9me3 are themselves differentially correlated with expression, with H3K9me2 being permissive to transcription and H3K9me3 repressive (Jih et al. 2017). Taken together, these results point to the importance of combinations of histone marks to regulation (i.e. the presence of a “histone code”), but also highlight the evolvability of this code depending on clade and genomic context. Future work on various organisms is required to elucidate how different combinations of histone marks shape gene expression, and the evolutionary and mechanistic pressures driving divergent patterns.

### Direct Evidence for the “Open in Same-Sex/Closed in Opposite” Model

If chromatin is associated with sex-specific regulation, sex-biased genes should show sex-specific chromatin marks. In particular, an excess of male-biased genes should be in open chromatin in males but in closed chromatin in females, and vice-versa (Brown and Bachtrog 2014). Brown and Bachtrog (2014) tested this directly in *Drosophila* by comparing the proportion of such sex-inconsistent genes among sex-biased and unbiased genes and found no evidence supporting this hypothesis (Brown and Bachtrog 2014). Recently, Nanni et al. (2023) found that male-biased genes in *Drosophila* heads were enriched in male-open chromatin, and depleted from female-open chromatin (while female-biased genes were enriched in female-open chromatin) (Nanni et al. 2023). However, they did not directly test for a reversal of chromatin state of the same gene between the sexes and were generally limited in the characterization of open and close chromatin by the relatively small number of marks used (one active, H3K4me3, and two repressive, H3K27me2/H3K27me3). Here, we were able to classify our genes as having active or repressed chromatin using 7 different histone marks, and in both heads and gonads of males and females. Very few genes were sex-biased in heads, unlike in the *D. melanogaster* study (Nanni et al. 2023). This is likely due to methodological differences, as Nanni et al. (2023) inferred sex-biased expression at the level of individual exonic features, whereas, in *Artemia*, sex-biased genes were identified based on the cumulative expression levels across all exons of a gene, and only if they had at least a two-fold change, a much more conservative approach. This precluded the detection of very subtle changes between the sexes, which typically comprise the bulk of sex-biased genes in *Drosophila* head and brain datasets (Huylmans and Parsch 2015). Whether such small changes are biologically relevant is not clear, and we therefore focused on more robust sex-biased expression, which is largely found in gonads. Our detailed classification showed that many genes have sex-inconsistent epigenetic states in the gonad, and these are enriched among sex-biased genes in the predicted direction. This provides a more direct test of the model than previously possible and strongly supports a contribution of the chromatin landscape to sex-specific regulation of expression.

### *Artemia franciscana* Exhibits *Drosophila*-like Dosage Compensation

Early large-scale gene expression studies highlighted an apparent discrepancy between ZW and XY species, with only the latter showing dosage compensation at the chromosome level (Gu and Walters 2017). Since then, multiple studies in Lepidoptera have shown that dosage compensation can regulate whole Z chromosomes, but this has so far remained exceptional among ZW systems (Walters, Hardcastle, and Jiggins 2015; Gu, Walters, and Knipple 2017). *A. franciscana* shows a significant enrichment of H4K16ac on the Z-specific regions of the Z chromosome compared to autosomes in females across both head and gonad tissues, consistent with dosage compensation acting to increase gene expression of the female Z. It should be noted that because of the small number of genes in the S0 (∼1% of all genes), subtler changes in other chromatin marks may not be as easily detected and cannot be fully excluded. The female enrichment in H4K16ac is in line with earlier results on gene expression, which showed that Z-specific and autosomal genes have similar expression levels in both males and females (Huylmans et al. 2019; Bett et al. 2024). This mechanism resembles the mode of action of dosage compensation in *Drosophila*, where the dosage compensation complex deposits active H4K16ac histone marks on the single male X-chromosome (Lucchesi and Kuroda 2015). Another interesting parallel is the presence of a candidate motif that is enriched in the differentiated region of the Z and may be targeted by the dosage compensation machinery, as suggested by its frequent co-occurrence with H4K16ac peaks. This is reminiscent of the high-affinity sites of *D. melanogaster*, which are enriched for a guanine-adenine (GA)-rich motif that allows for their recognition and binding by the dosage compensation complex (Alekseyenko et al. 2008; Straub et al. 2008). H4K16ac-mediated dosage compensation has also been observed in green anole lizards, where males exhibit an enrichment of H4K16ac on their X-chromosome (Marin et al. 2017), although in this case recognition and binding to the X-chromosome is mediated by a long non-coding RNA (Tenorio et al. 2024). Contrary to the original dichotomy between ZW and XY systems, these results therefore not only suggest that chromosome-wide compensation of Z chromosomes may be more common than previously thought, but also highlight a striking case of molecular convergence of the regulation of the 2 types of sex chromosomes. Future work in diverse ZW and XY systems is needed to shed light on how common different types of compensation are, and what evolutionary scenarios lead to each of them.

## Methods

### Dissection of Heads and Gonads of *Artemia franciscana*

*Artemia franciscana* cysts were purchased by the Fish Facility of the Institute of Science and Technology Austria from the Zierfischfutter Wünnenberg (who source them from Sanders), and hatched at 24 to 26 °C, in 550 ml red sea salt in 24L in 14:10 h light/darkness. Freshly hatched nauplii were transferred to the lab and reared until adulthood in individual vials to avoid mating under 50 grams/litres salinity and in 16:08 h light/darkness. Salt was washed off adult *A. franciscana* with deionized water and 1 × PBS (without MgCl_2_ and CaCl_2_), and tissues were dissected under cold DPBS. The ovisac contents from the female gonads were emptied and discarded to minimize the effect of the female germline on our results, as germ cells have recently been shown to lack dosage compensation in *A. franciscana* (Elkrewi and Vicoso 2024). Tissues from 4 adults per replicate were then permeabilized for 30 min in an ice-cold mixture of 10 mg of Collagenase and 1 ml of Enzyme Dissociation Buffer.

### CUT&Tag Library Preparation

CUT&TAG assays were performed using CUT&Tag-IT™ Assay Kit (Active Motif cat no. 53160). Permeabilized tissues were mixed with activated Concanavalin A beads after their resuspension in the wash buffer. We then incubated overnight at 4 °C with primary antibodies from Active Motif: H3K27me3 (cat no. 39157), H3K36me3 (cat no. 61102), H3K4me3 (cat no. 39160), H3K27ac (cat no. 39034), H4K16ac (cat no. 39930), H3K9me3 (cat no. 39766) and H3K9me2 (cat no. 39754). Following this, samples were incubated with a diluted Guinea Pig Anti-Rabbit secondary antibody for 1 h in a nutator at room temperature. The beads were then washed with a Dig-Wash buffer, and assembled pA-Tn5 transposomes were then added and incubated at room temperature for 1 h in a nutator mixer. Following incubation, washing of the beads was performed using a Dig-300 buffer. Tagmentation was completed by adding a tagmentation buffer that was mixed with Protease Inhibitor Cocktail and 5% Digitonin and then incubating them at 37 °C for 80 min. In order to halt the tagmentation process and digest DNA fragments, 4.2 μl of EDTA, 1.25 μl of 10% SDS, and 1.1 μl of Proteinase K were added to the Tagmentation buffer and incubated for 60 min at 55 °C. DNA was extracted and then amplified in a 50 μl PCR reaction using a combination of unique i7 Indexed primer and one i5 Indexed primer, 30 ul of tagmented DNA, 1 ul of 10 mM dNTPs, 10 ul of 5× Q5 Reaction Buffer, and 0.5 ul of Q5 DNA polymerase. The desired fragment size libraries of CUT&TAG were then selected using 1.1 × of solid-phase reversible immobilization (SPRI) beads to DNA (55μl of SPRI was added to 50 μl of DNA). The libraries were checked for quality using both Agilent Bioanalyzer for fragment size distribution and qPCR for DNA library concentration, and those that passed minimum quality checks (with at least 2.5 nM in concentration) were sequenced on Illumina NextSeq550 PE150 Medium at the Vienna Biocenter sequencing facility.

### Processing of CUT&TAG Raw Reads

Paired-end raw reads were checked for quality with fastqc (Andrews 2010). Overrepresented sequences were filtered out using the bbduk.sh package of bbmap tool (Bushnell 2014) with the options “ref = overrepresented.fasta ktrim = r k = 31 mink = 11 hdist = 1 tpe tbo.” Sequencing adapters were then trimmed with cutadapt under parameters “-a CTGTCTCTTATACAC -A CTGTCTCTTATACAC -O 5 -q 0 -m 20 -j 30” (Martin 2011). The trimmed paired-end reads were aligned to *A. franciscana* reference genome (Bett et al. 2024) using bowtie2 v2.4.5 (Langmead and Salzberg 2012) with these options: “–local –very-sensitive-local –no-unal –no-mixed –no-discordant –phred33 -I 10 -X 700.” The alignments were sorted by coordinates using Picard (“Picard Toolkit” 2019) with the parameter “-SORT_ORDER coordinate.” PCR duplicates were then marked and removed with Picard using the options “REMOVE_DUPLICATES = true, ASSUME_SORTED = TRUE.” The resulting alignments were filtered further with the options “-q 2 -F 0 × 04 -f 0 × 2” in samtools v1.18 (Li et al. 2009) and used for downstream analysis (supplementary table S7, Supplementary Material online). Consistency between biological replicates was addressed by calculating the pairwise Pearson correlation coefficient of the normalized coverage along TEs and genes, for all of the samples using the R function “cor” from the package stats (supplementary fig. S20, Supplementary Material online).

### Expression Analysis

The RNA-seq samples that were obtained from the heads and gonads of 10 adult males and 10 adult females of *A. franciscana* (Huylmans et al. 2019) were mapped (supplementary table S8, Supplementary Material online) using Kallisto (Bray et al. 2016) to the annotated coding sequences (CDs) of *A. franciscana* (from annotation (Bett et al. 2024)) to create pseudoalignment and were then imported to sleuth (Pimentel et al. 2017) for estimation of transcript abundance. The gene expression within each tissue across sexes was normalized using NormalyzerDE (Willforss, Chawade, and Levander 2019) in Rv4.1.2. We then performed differential expression analysis with the sleuth package using a likelihood ratio test (LRT). Genes that exhibited more than 2-fold change (FC) difference with a false discovery rate (FDR) of <0.05 in expression were categorized as male or female-biased depending on the direction of the change.

### Identification of Optimal Chromatin State Number

We used Spectacle to assign chromatin states along the *A. franciscana* genome (Song and Chen 2015). First, alignment of CUT&TAG reads from each of the 7 histone marks was converted to bed format with bedtools and then binarized across the genome in a bin size of 200 bp using BinarizedBed with option “-strictthresh.” Second, binarized data were then used in the learning model of each given chromatin state number. This was performed with LearnModel with the options ‘-i spectral -comb’ to allow the hidden Markov model to use a spectral learning algorithm in its chromatin state estimation. Next, we employed the CompareModels function within Spectacle to assess the maximum emission parameters, correlating them with selected chromatin states using Pearson correlation. Specifically, we examined the correlation of chromatin state 21 with a range of simpler models, encompassing chromatin state numbers from 20 to 2. The median of these correlations was plotted to pinpoint the optimal chromatin state number (supplementary fig. S1, Supplementary Material online). We identified 12 chromatin states as the optimal state number, as any further increase in the number of chromatin states only lead to the recovery of states with similar characteristics to the starting chromatin state 21. Therefore, chromatin state number 12 was selected for downstream analysis.

### Analysis of Chromatin States

The coverage of each emission state (chromatin state 12) across the *A. franciscana* genome was generated using the MakeSegmentation function. The outputs were then intersected with the coordinates of genes using the bedtools intersect function under default settings. Emission states were further summarized into 3 major groups depending on their functional annotation (Brown and Bachtrog 2014); (i) active transcription (H3K27ac, H4K16ac, H3K4me3, and H3K36me3), (ii) polycomb-mediated repression (H3K27me3) and (iii) constitutive heterochromatin (H3K9me3 and H3K9me2). Additionally, the inclusion of mixed states that contain combination of either facultative or constitutive histone marks (H3K9me3, H3K9me2, and H3K27me3) with any of the active transcription marks (H3K36me3, H3K27ac, H3K16ac, and H3K4me3) and Null state resulted in definition of 16 chromatin signatures as done in (Gueno et al. 2022).

### Correlating Genes With Chromatin Marks

To assess the enrichment of chromatin marks across genes, we adopted a similar approach to that of Anderson et al. (2023) by tallying the mapped reads spanning from the beginning to the end of every gene in each replicate. This was accomplished by aggregating reads with deepTools/multiBamSummary using a bed annotation file and then normalizing counts based on the number of mapped reads and the gene length in kilobases in Million. The resulting metric provides counts per kilobase per million (CPKM).

“*multiBamSummary BED-file –BED annotation.bed –bamfiles alignment.bam –centerReads –maxFragmentLength 700 -o results.npz –outRawCounts readCounts.tab*”

To generate average plots depicting chromatin signals surrounding the TSS of genes of each replicate, we applied deepTools/bamcoverage to summarize normalized coverage within a window of 10-bp intervals “*bamCoverage -b alignment.bam –binSize 10 -p 20 –normalizeUsing RPKM –maxFragmentLength 700 -o file.bw*”.

To average the distribution of chromatin marks of surrounding TSS of genes in both replicates, we used bamCompare function of deepTools “*bamCompare –bamfile1 file1.bam –bamfile2 file2.bam -p 20 -bs 5 –normalizeUsing RPKM –skipNAs –minFragmentLength 10 –maxFragmentLength 700 –operation mean –scaleFactorsMethod None -o output.bw.*”

The outputs were used as inputs for computing matrices using deepTools/computeMatrix “*computeMatrix reference-point –referencePoint TSS -S file.bw -R annotation.bed –missingDataAsZero -b 1000 -a 2000 –binSize 5 -o file.mat.gz*” and visualized using plotProfile feature.

In order to visualize the distribution of H4K16ac in each tissue across all 21 chromosomes of *A. franciscana*, we used genome coverage data generated using deepTools/bamCoverage with these options: “*bamCoverage -b file.bam -bs 30000 -p 20 –outFileFormat bedgraph –normalizeUsing RPKM –maxFragmentLength 700 -o file.bedgraph.*”

### Peaks Calling and Motif Enrichment

The deduplicated alignments generated earlier were converted to BED format using the bamToBed function from Bedtools (Quinlan and Hall 2010). Read pairs mapping to the same chromosome with fragment lengths under 700 bp were selected for the generation of bedgraph files with the genomecov function in Bedtools (Quinlan and Hall 2010). Peaks were then called using SEACR [Sparse Enrichment Analysis for CUT&RUN v1.3 (Meers, Tenenbaum, and Henikoff 2019)]. The SEACR peaks were called using “SEACR_1.3.sh sample.bedgraph 0.01 non stringent sample_seacr_top0.01.peaks.” The H4K16ac peaks corresponding to selected genes (TPM > 0.5) of Z-specific region (S0) in females were used for sequence motifs identification using HOMER (Hypergeometric Optimization of Motif EnRichment (Heinz et al. 2010)). Motifs were identified as follows; “findMotifsGenome.pl H4K16acpeaks.bed Afranciscana_genome.fa.masked S0peaks_homerMotif -mask -size 1000 -p 20.” In order to identify instances of the targeted motif of interest, we used scanMotifGenomeWide.pl function of HOMER as follows: scanMotifGenomeWide.pl S0peaks_homerMotif/homerResults/motif1.motif Afranciscana_genome.fa.masked -mask -keepAll -bed > S0peaks_homerMotif/motif1.sites.bed

### Estimating Rates of Evolution of Genes With and Without H4K16ac Peaks

The CDS of annotated genes from *Artemia franciscana* (Bett et al. 2024) and their homologous sequences of annotated genes from *Artemia sinica* (Elkrewi et al. 2022) were aligned using the TranslatorX software package (Abascal, Zardoya, and Telford 2010) with the “gblocks” option to filter out poorly aligned or unreliable regions, ensuring a high-quality alignment for downstream analyses. Following the alignment, the nonsynonymous (Ka) and synonymous (Ks) substitution rates were calculated using the KaKs_calculator (Wang et al. 2010), employing the Yang and Nielsen (YN) algorithm.

## Supplementary Material

msaf085_Supplementary_Data

## Data Availability

The CUT&TAG raw reads generated in this study have been deposited in the National Center for Biotechnology Information (NCBI) BioProject under accession number PRJNA1150095. The Bioinformatics pipeline that was used for processing and analysis of CUT&TAG sequencing reads can be accessed on this gitpage (https://github.com/vkb25/Chromatin-landscape-in-Artemia-franciscana.git).
