# Supplementary material for: Chromatin Landscape Is Associated With Sex-Biased Expression and Drosophila-Like Dosage Compensation of the Z Chromosome in Artemia franciscana
Source: Mol Biol Evol. 2025 Apr 9;42(5):msaf085. doi: 10.1093/molbev/msaf085 (PMC12060005; doi:10.1093/molbev/msaf085)
Supplement: msaf085_Supplementary_Data [file msaf085_supplementary_data.pdf]

Supplementary Information

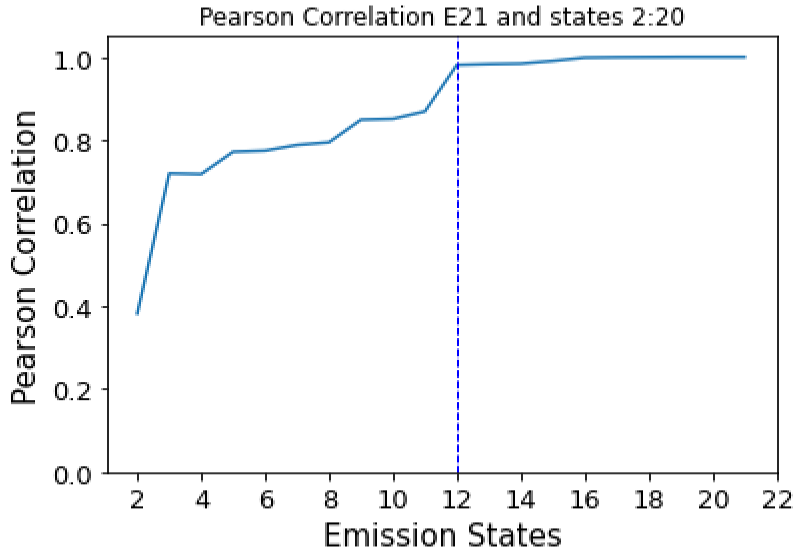

Supplementary Figure 1: Pearson Correlation of selected model (E21) to sets of models (E20:E2) to identify optimal number of chromatin states.

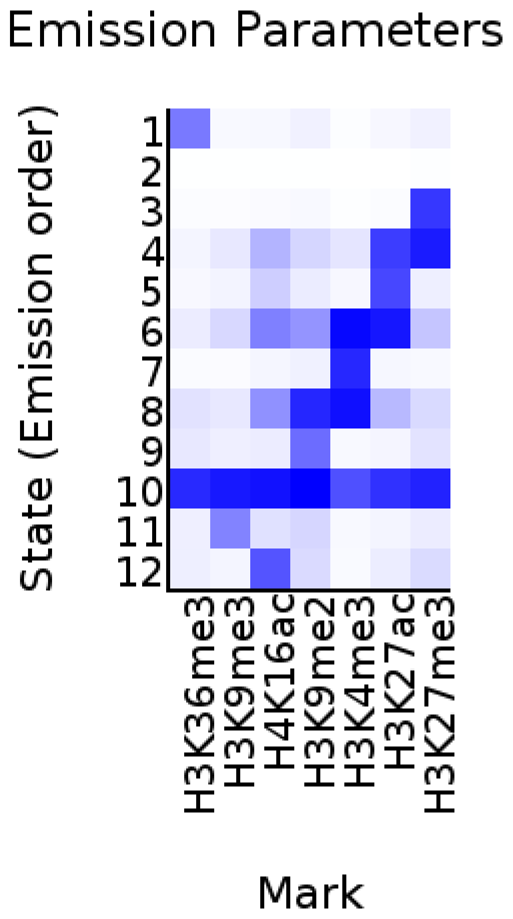

Supplementary Figure 2: Emission model showing likelihood of finding histone mark in each chromatin state number. The darker the color means higher chance of presence of that particular histone mark.

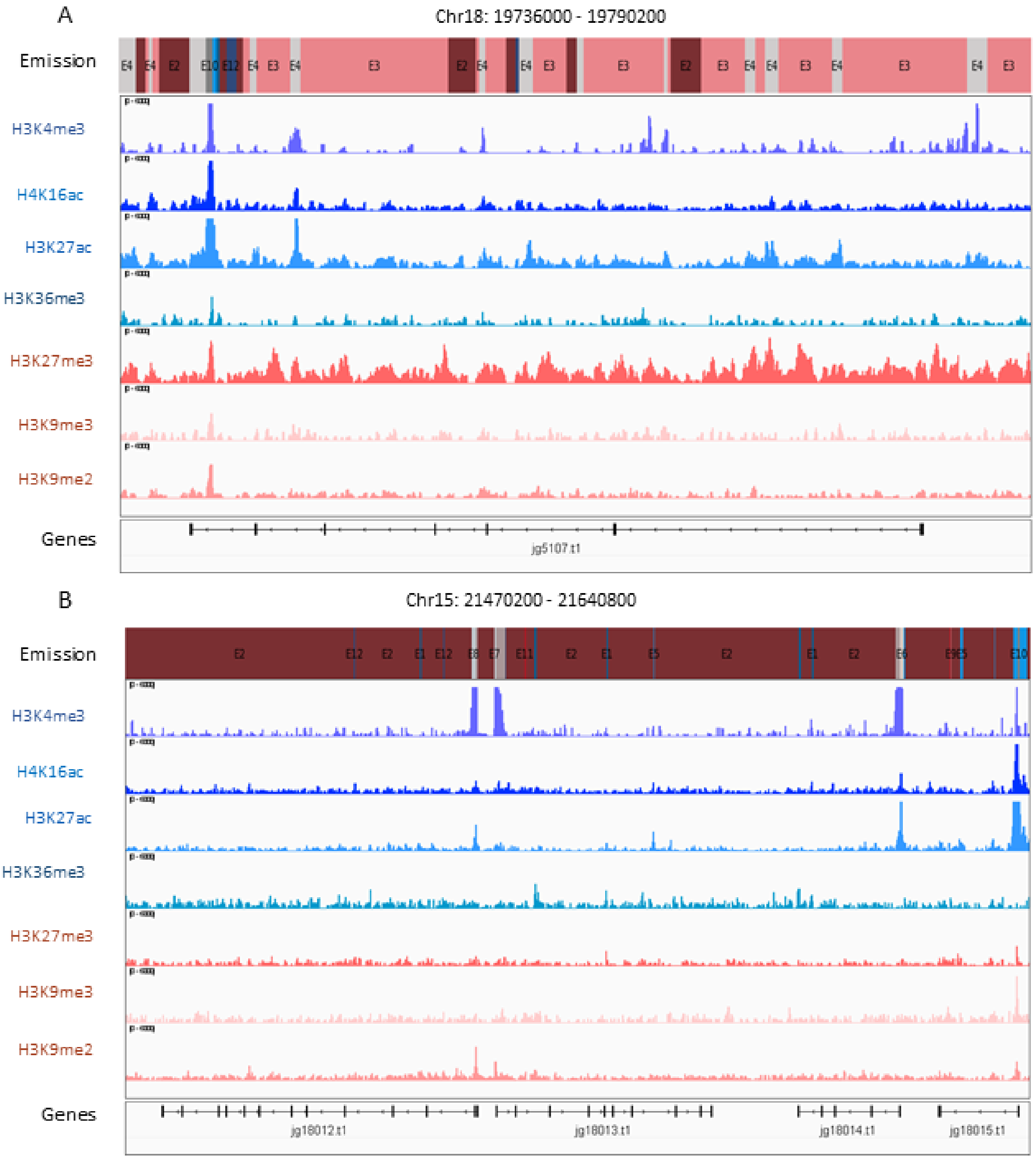

Supplementary Figure 3: Representative emission state profiles for genomic regions of chromosome 18 (A) and chromosome 15 (B) in male head tissue.

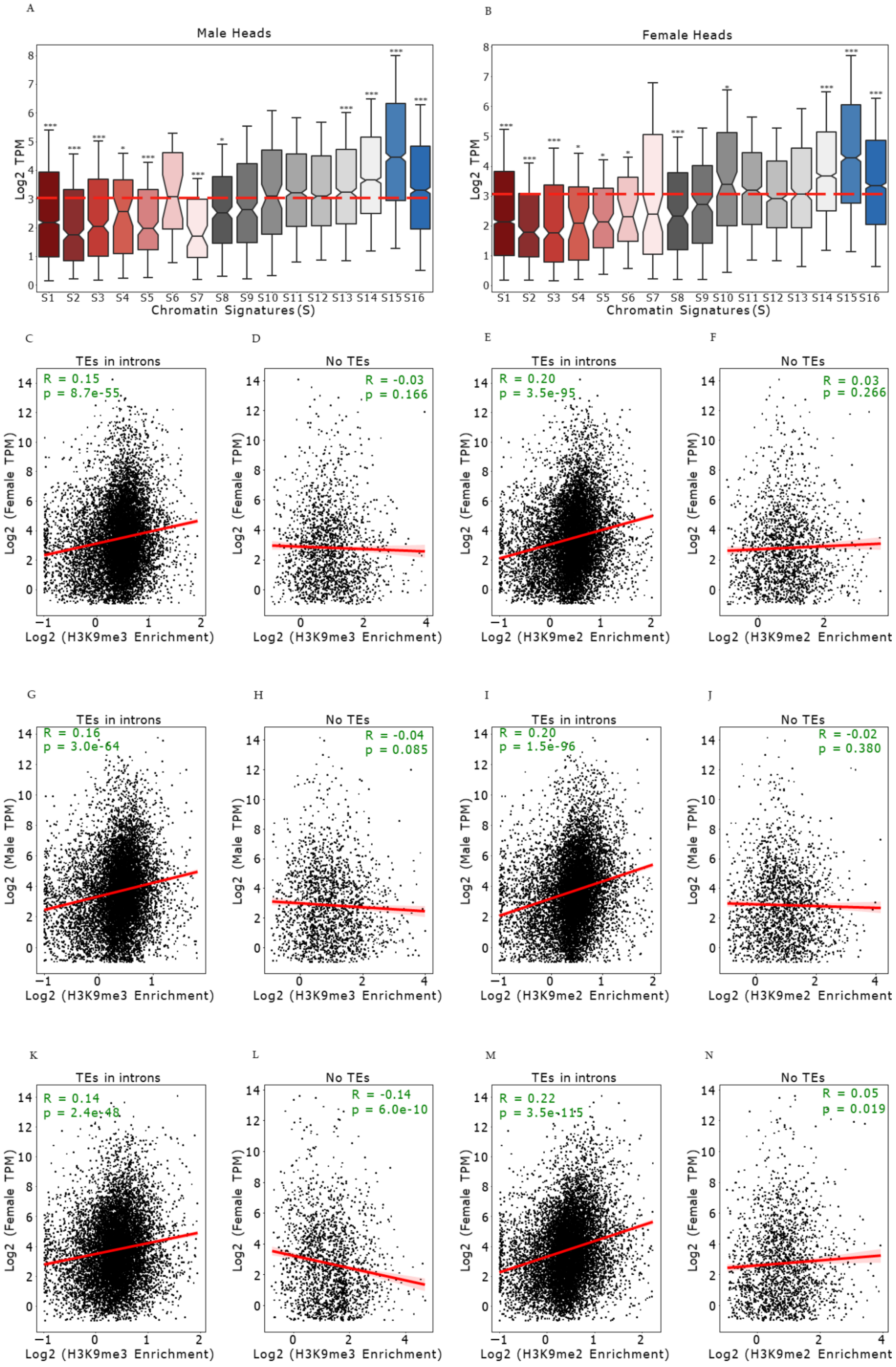

A
Male Heads
Log2 TPM
Chromatin Signatures (S)
B
Female Heads
C
TEs in introns
R = 0.15
p = 8.7e-55
Log2 (Female TPM)
Log2 (H3K9me3 Enrichment)
D
No TEs
R = -0.03
p = 0.166
E
TEs in introns
R = 0.20
p = 3.5e-95
Log2 (H3K9me2 Enrichment)
F
No TEs
R = 0.03
p = 0.266
G
TEs in introns
R = 0.16
p = 3.0e-64
Log2 (Male TPM)
H
No TEs
R = -0.04
p = 0.085
I
TEs in introns
R = 0.20
p = 1.5e-96
J
No TEs
R = -0.02
p = 0.380
K
TEs in introns
R = 0.14
p = 2.4e-48
L
No TEs
R = -0.14
p = 6.0e-10
M
TEs in introns
R = 0.22
p = 3.5e-115
N
No TEs
R = 0.05
p = 0.019

12

Supplementary Figure 4: Expression of chromatin signatures (W and Z-specific genes were excluded) in male (A) and female heads (B). Panels C to D show the correlation of female heads' expression with H3K9me3 for genes with (C) or without (D) TEs in their introns. Panels G and H show the same for male gonads expression. Panels K and L show the same for female gonads expression. Panels E and F show the correlation of female heads' expression with H3K9me2 for genes with (E) or without (F) TEs in their introns. Panels I and J show the same for male gonads expression. Panels M and N show the same for female gonads expression.

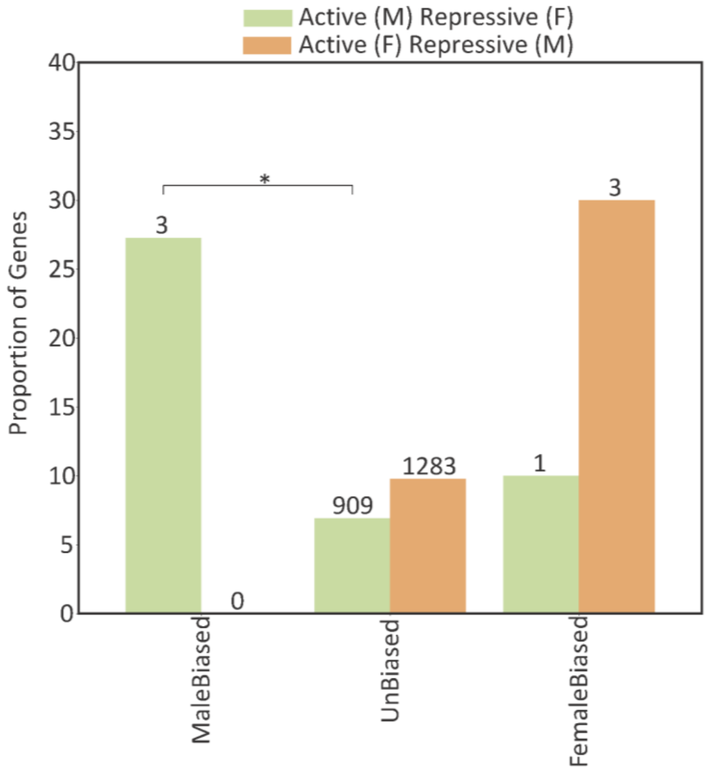

Supplementary Figure 5: Proportion of genes with contrasting chromatin states in males and females and which have sex-biased expression in heads of *A. franciscana* (FDR <0.05, Foldchange >2 & TPM >0.1 for genes in *A. franciscana*)

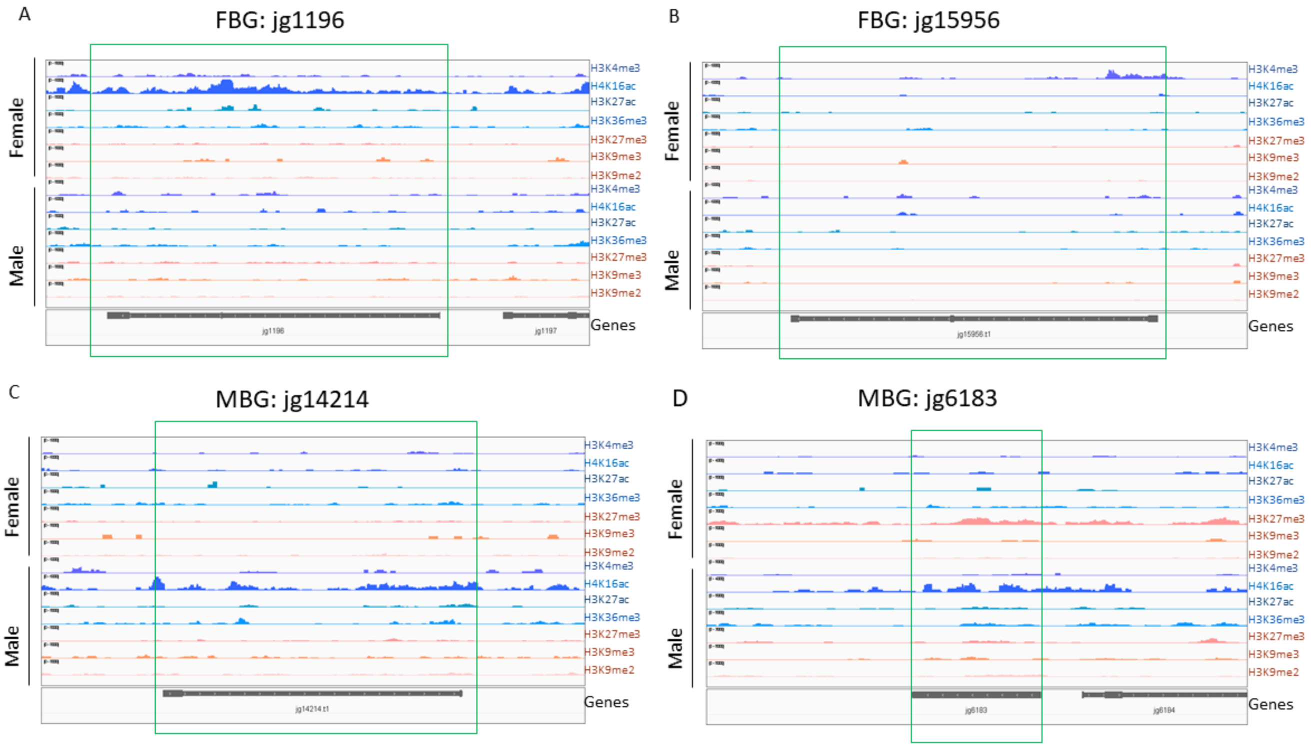

Supplementary Figure 6: **Representative chromatin profile regions for female-biased genes** (A and B) on chromosome 20 (jg1196; is enriched with H4K16ac (E12, chromatin signature S16) in females and null states in males E2 (S1) and chromosome 17 (jg15956; is enriched with H3K4me3 (E7, chromatin signature S16) in females and null states E2 (S1) in males). **Representative chromatin profile regions for male-biased genes** (C and D) on chromosome 7 (jg14214; is enriched with H4K16ac (E12 chromatin signature S16) in males and null states in females E2 (chromatin signature S1) and chromosome 16 (jg6183; is enriched with H4K16ac (E12, chromatin signature S16 in males) while in female is enriched with H3K27me3 (E3, chromatin signature S2) .

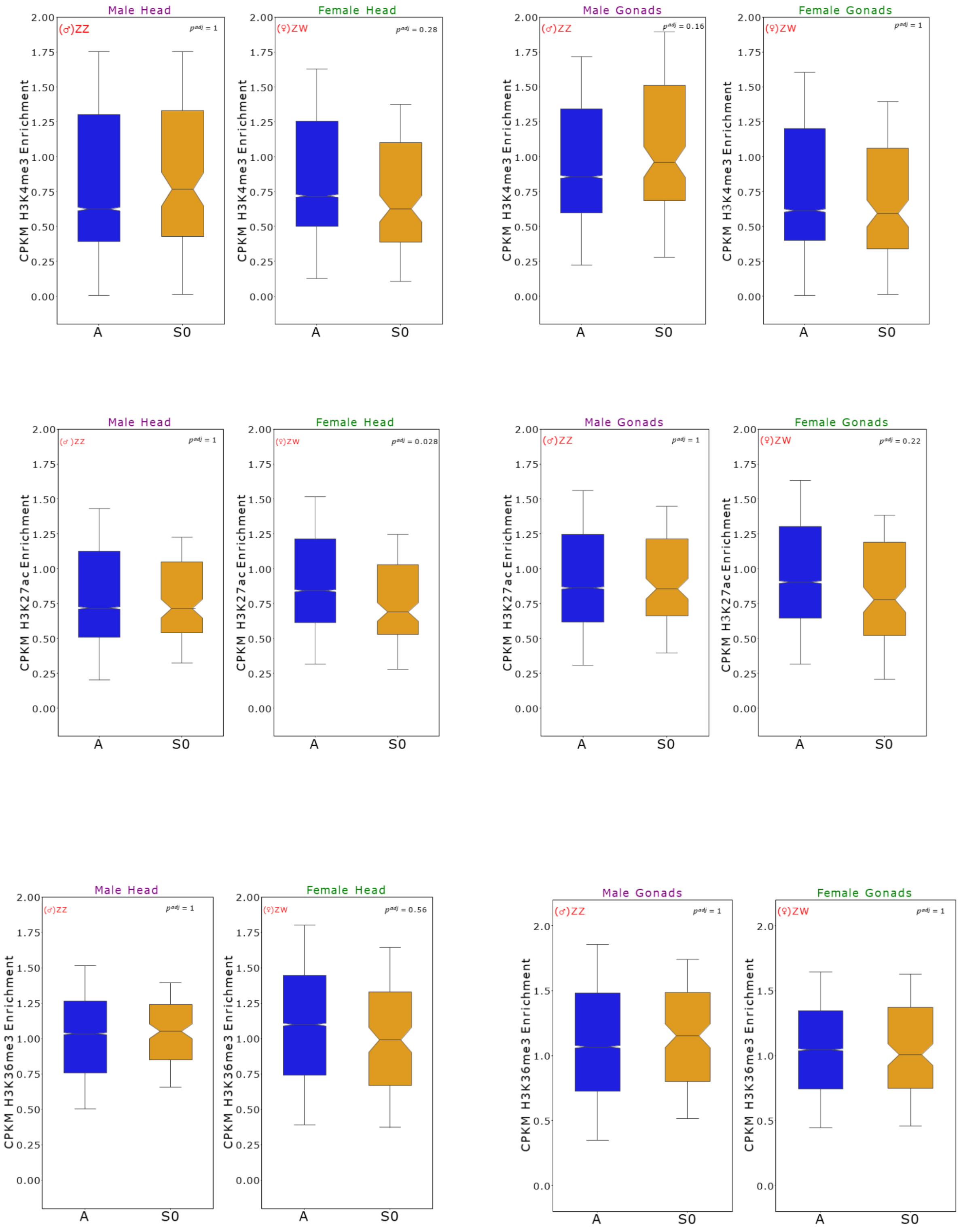

Supplementary Figure 7: Normalized CPKM enrichment of active associated chromatin marks based on autosomal and Z-specific genes in somatic and gonadal tissues of *A. franciscana*.

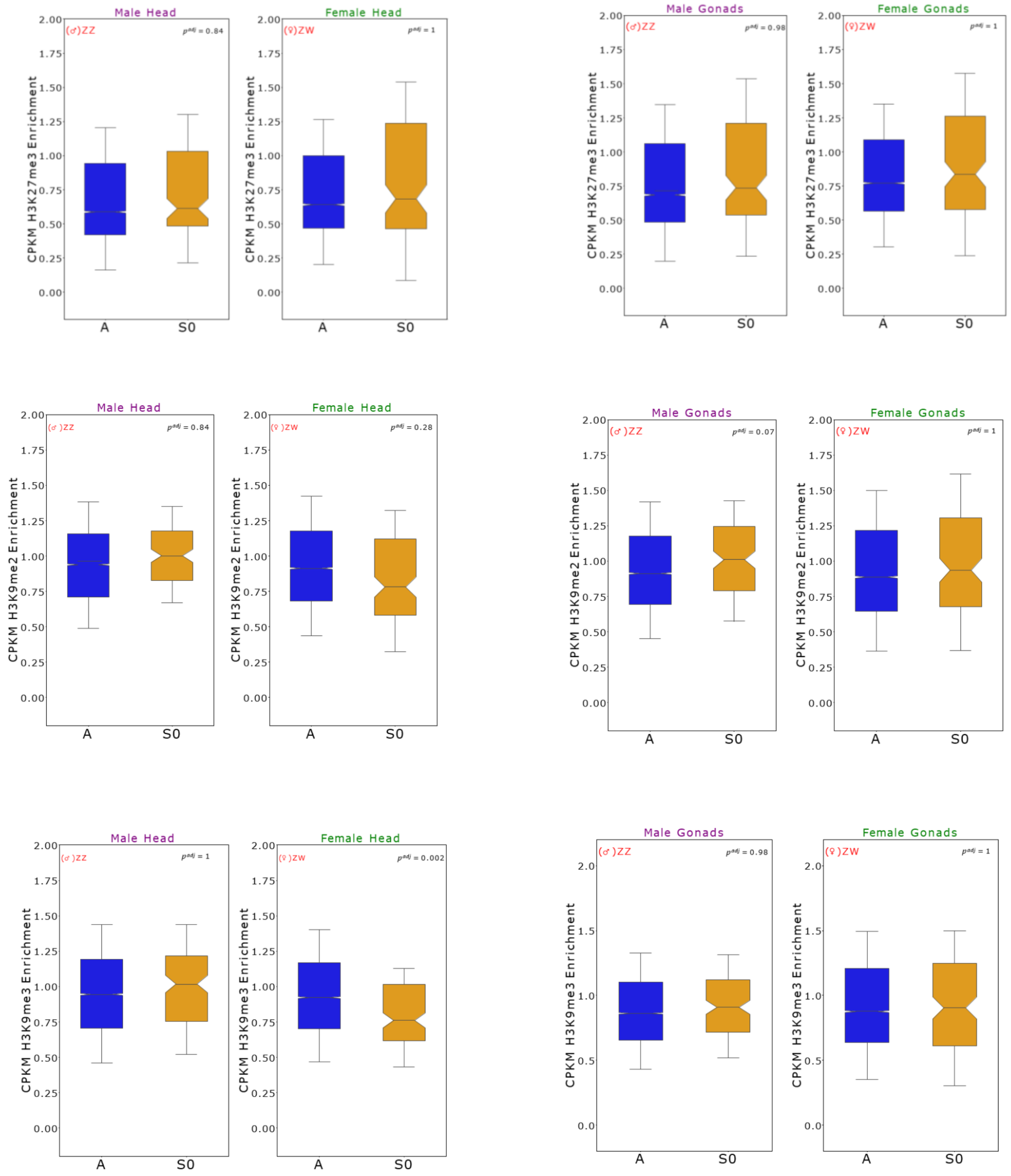

Supplementary Figure 8: Normalized CPKM enrichment of repressive associated chromatin marks based on autosomal and Z-specific genes in somatic and gonadal tissues of *A. franciscana*.

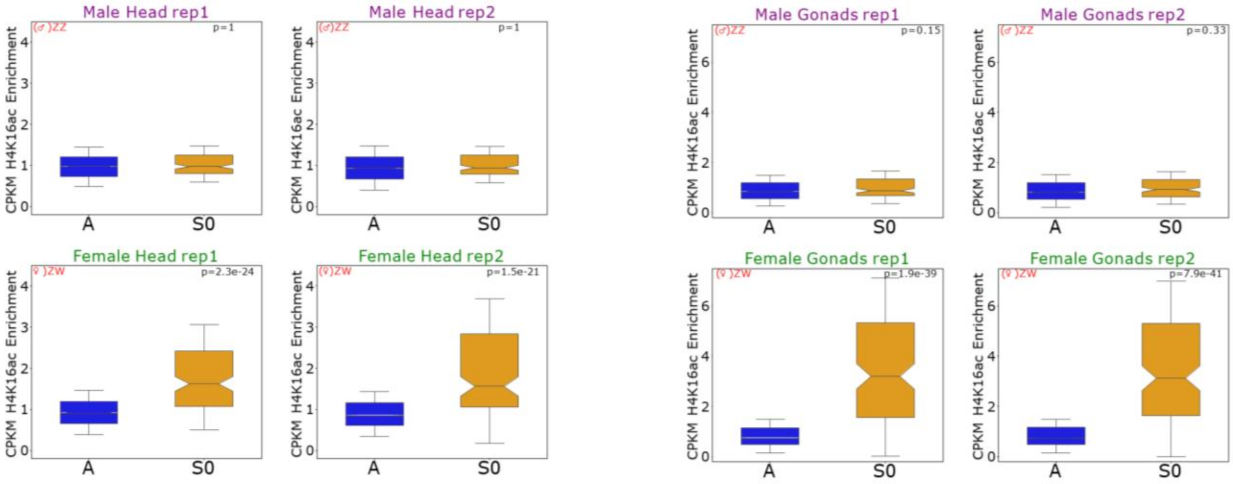
Male Head rep1
Male Head rep2
Male Gonads rep1
Male Gonads rep2
Female Head rep1
Female Head rep2
Female Gonads rep1
Female Gonads rep2
(♂)ZZ
(♀)ZW
p=1
p=0.15
p=0.33
p=2.3e-24
p=1.5e-21
p=1.9e-39
p=7.9e-41
CPKM H4K16ac Enrichment
A
S0
43

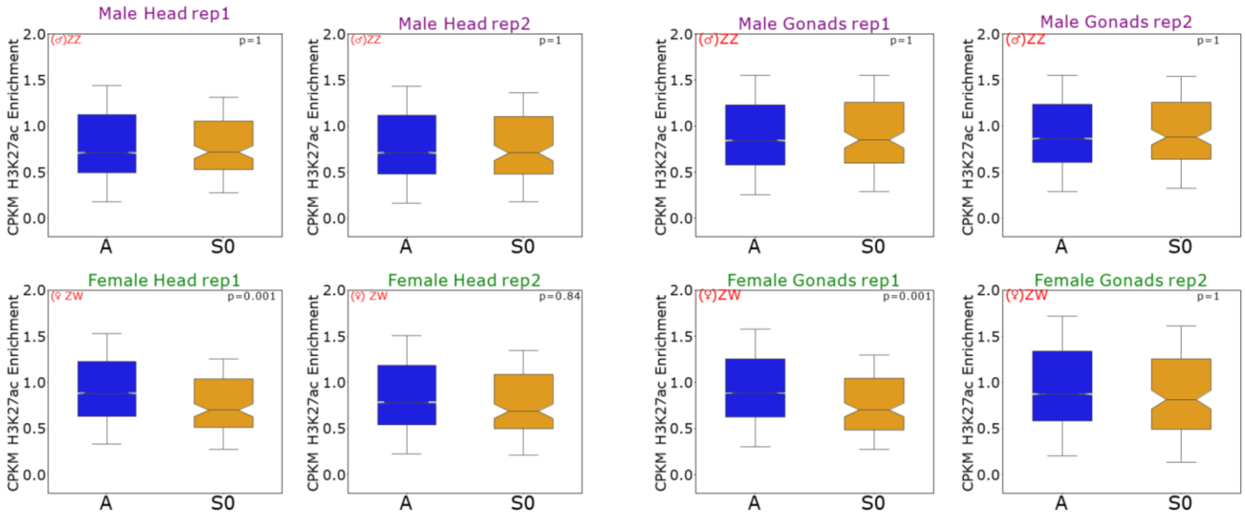
Male Head rep1
Male Head rep2
Male Gonads rep1
Male Gonads rep2
Female Head rep1
Female Head rep2
Female Gonads rep1
Female Gonads rep2
(♂)ZZ
(♀)ZW
p=1
p=0.001
p=0.84
CPKM H3K27ac Enrichment
A
S0
44

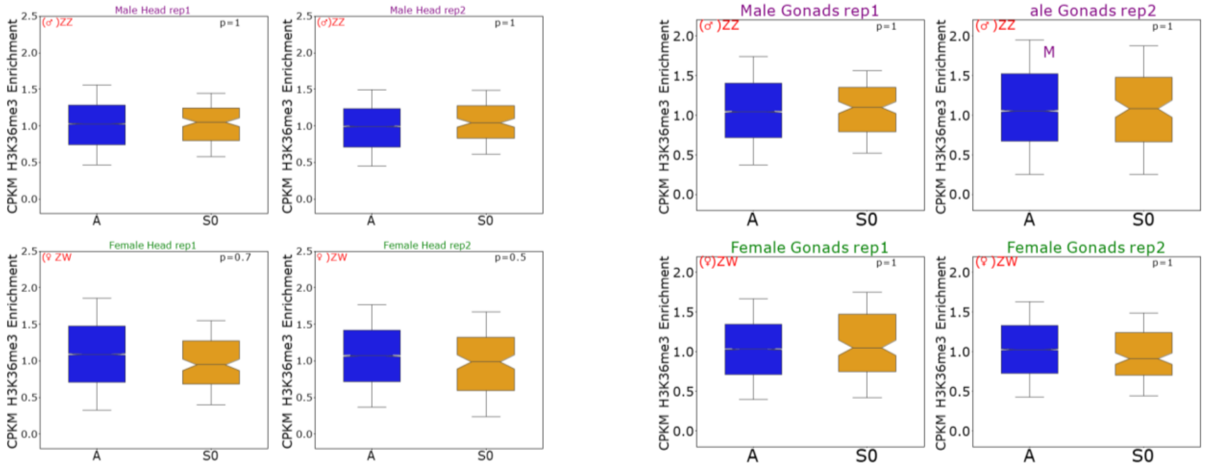
Male Head rep1
(♂)ZZ
p=1
Male Head rep2
(♂)ZZ
p=1
Male Gonads rep1
(♂)ZZ
p=1
ale Gonads rep2
(♂)ZZ
p=1
M
Female Head rep1
(♀)ZW
p=0.7
Female Head rep2
(♀)ZW
p=0.5
Female Gonads rep1
(♀)ZW
p=1
Female Gonads rep2
(♀)ZW
p=1
CPKM H3K36me3 Enrichment
A
S0

45

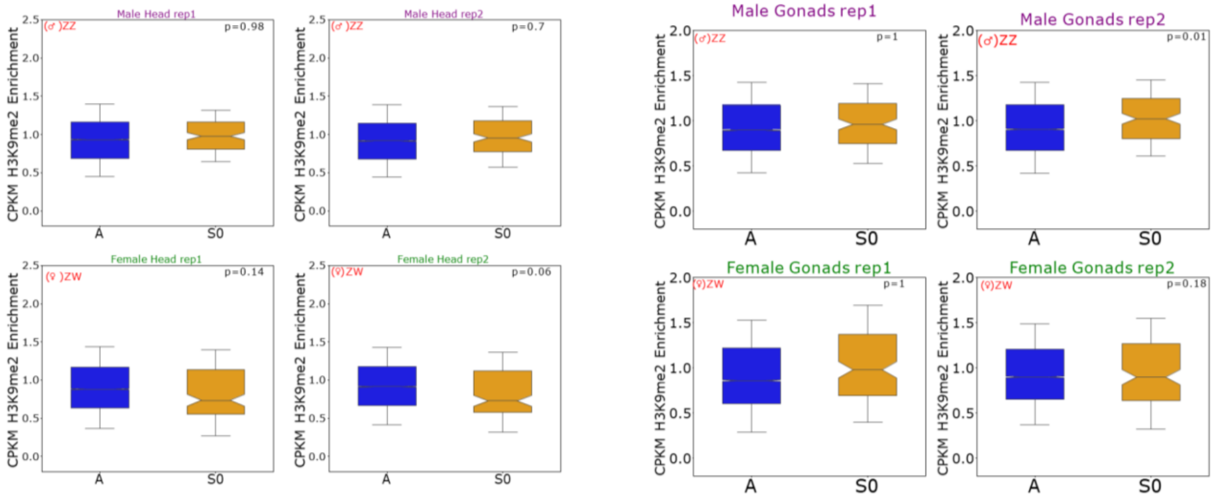
Male Head rep1
(♂)ZZ
p=0.98
Male Head rep2
(♂)ZZ
p=0.7
Male Gonads rep1
(♂)ZZ
p=1
Male Gonads rep2
(♂)ZZ
p=0.01
Female Head rep1
(♀)ZW
p=0.14
Female Head rep2
(♀)ZW
p=0.06
Female Gonads rep1
(♀)ZW
p=1
Female Gonads rep2
(♀)ZW
p=0.18
CPKM H3K9me2 Enrichment
A
S0

46

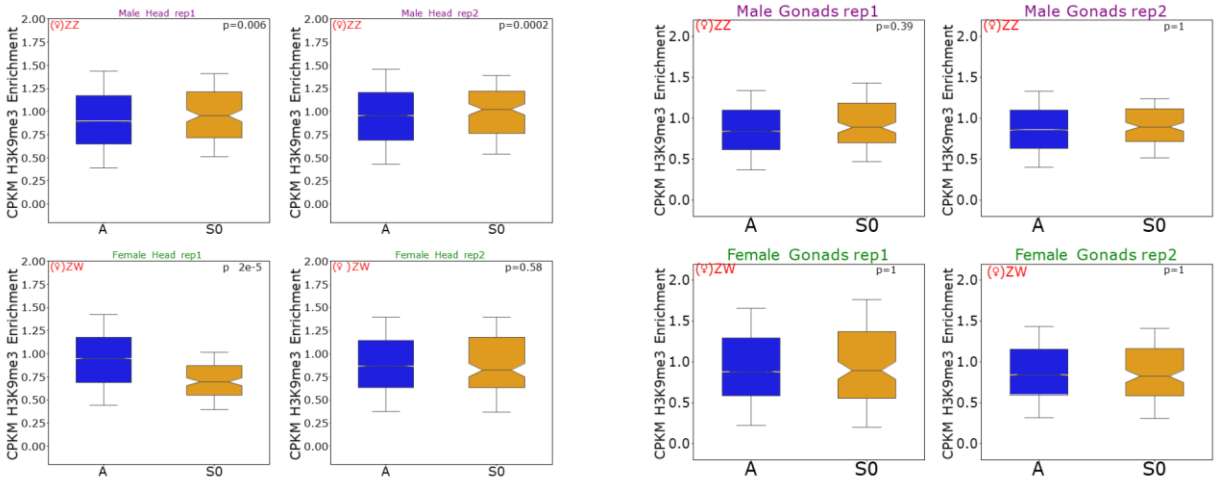
Male Head rep1
(♀)ZZ
p=0.006
Male Head rep2
(♀)ZZ
p=0.0002
Male Gonads rep1
(♀)ZZ
p=0.39
Male Gonads rep2
(♀)ZZ
p=1
Female Head rep1
(♀)ZW
p 2e-5
Female Head rep2
(♀)ZW
p=0.58
Female Gonads rep1
(♀)ZW
p=1
Female Gonads rep2
(♀)ZW
p=1
CPKM H3K9me3 Enrichment
A
S0

47

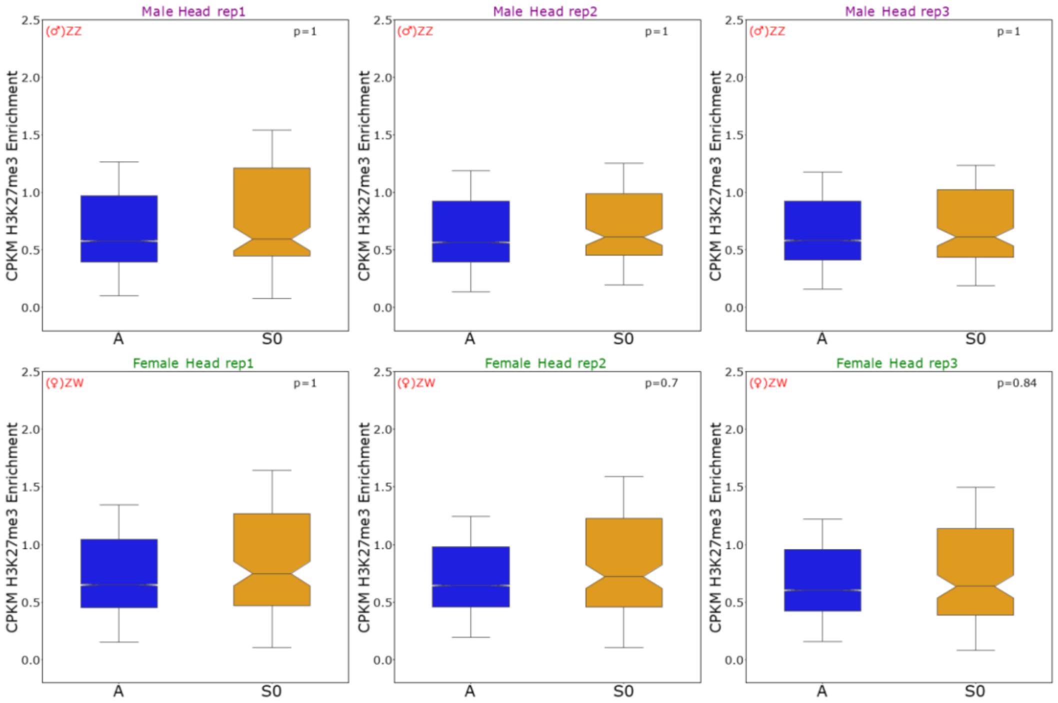
Male Head rep1
Male Head rep2
Male Head rep3
(♂)ZZ
p=1
CPKM H3K27me3 Enrichment
A
S0
Female Head rep1
Female Head rep2
Female Head rep3
(♀)ZW
p=1
p=0.7
p=0.84

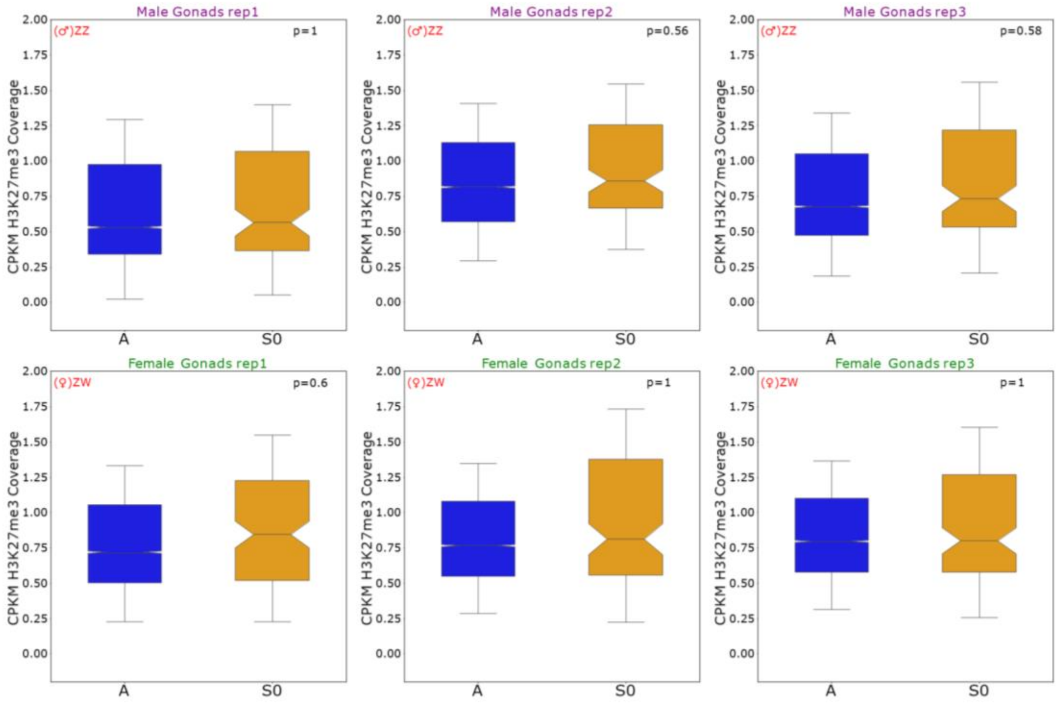
Male Gonads rep1
Male Gonads rep2
Male Gonads rep3
(♂)ZZ
p=1
p=0.56
p=0.58
CPKM H3K27me3 Coverage
A
S0
Female Gonads rep1
Female Gonads rep2
Female Gonads rep3
(♀)ZW
p=0.6
p=1

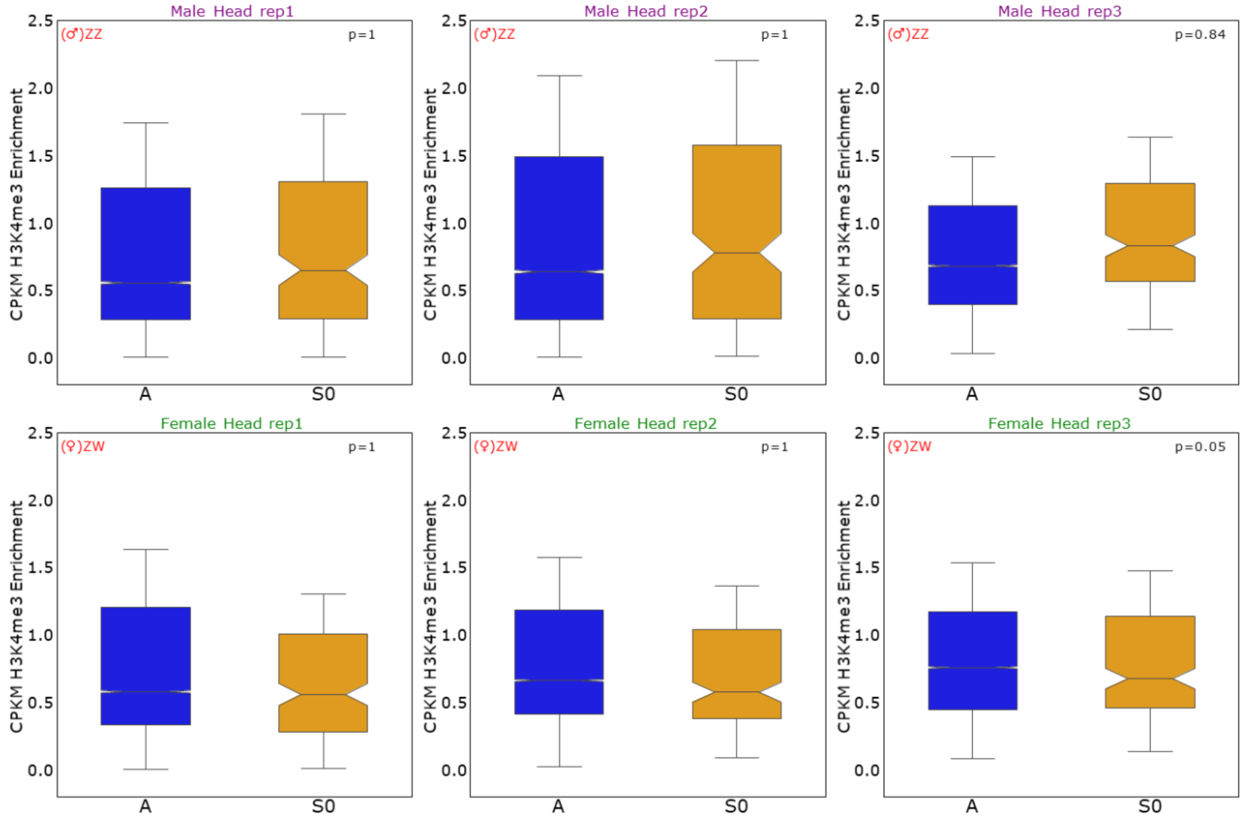
Male Head rep1
(♂)ZZ
p=1
Male Head rep2
(♂)ZZ
p=1
Male Head rep3
(♂)ZZ
p=0.84
Female Head rep1
(♀)ZW
p=1
Female Head rep2
(♀)ZW
p=1
Female Head rep3
(♀)ZW
p=0.05
CPKM H3K4me3 Enrichment
A
S0

50

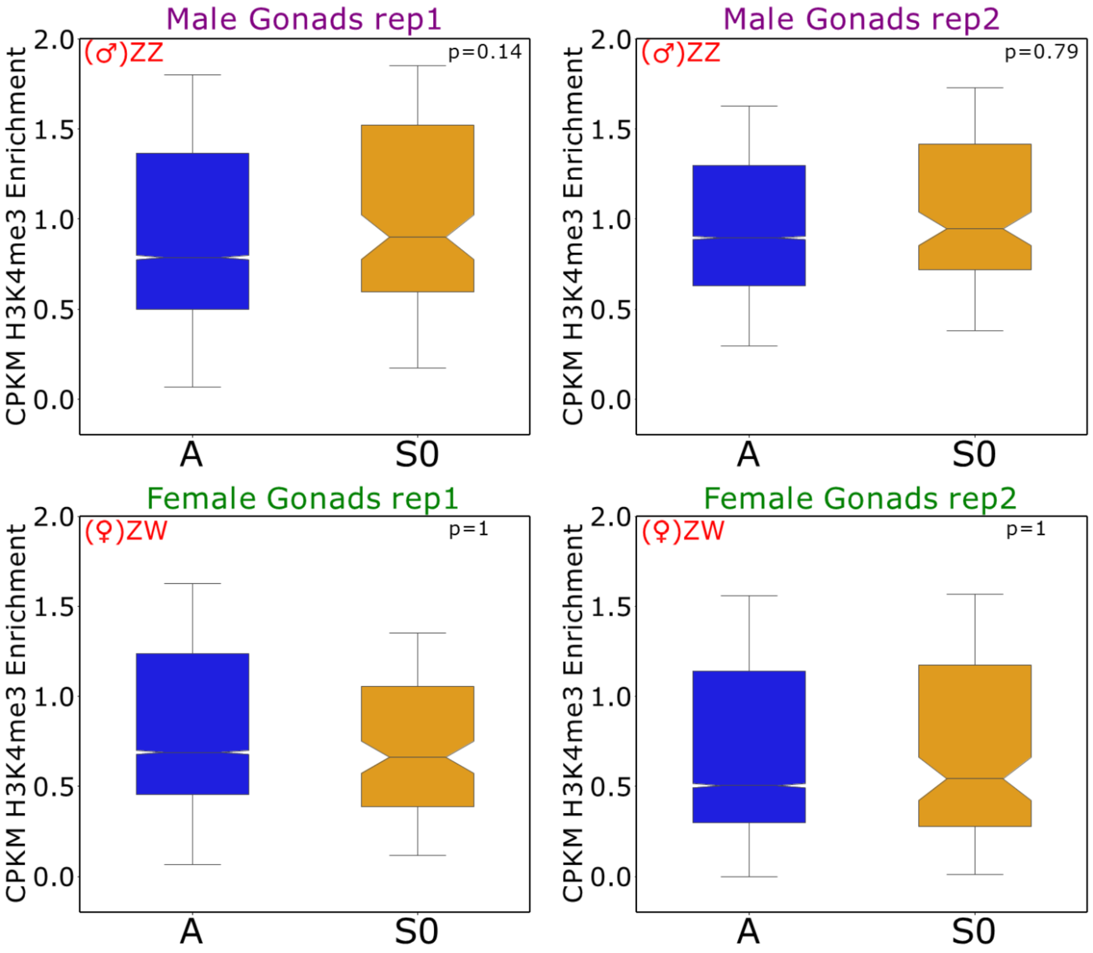

Supplementary Figure 9: Normalized CPKM enrichment of both active and repressive associated chromatin marks based on autosomal and Z-specific genes in each replicate in head and gonadal tissues of *A. franciscana*

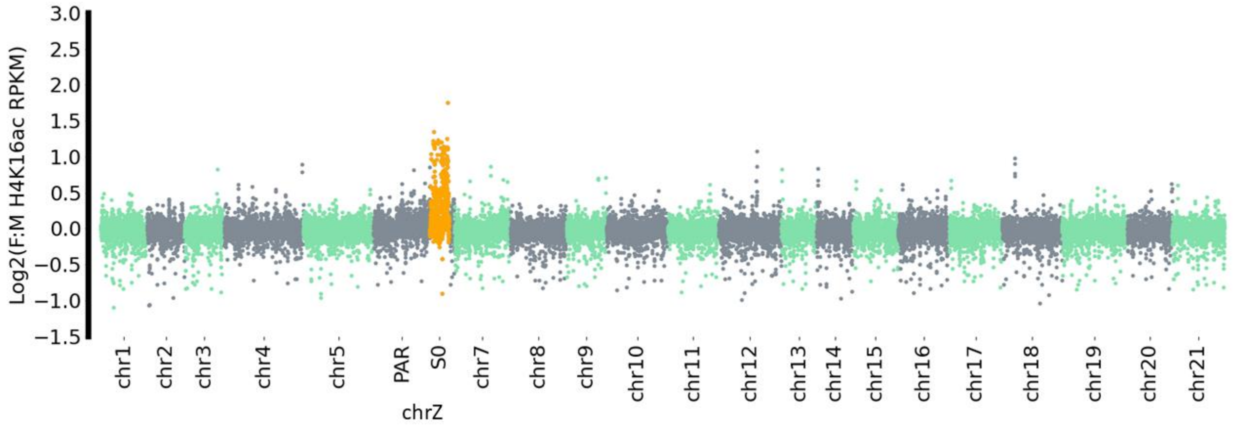

Supplementary Figure 10: Female to male coverage distribution of H4K16ac in 30kb windows across 21 chromosomes in Heads in *A. franciscana*.

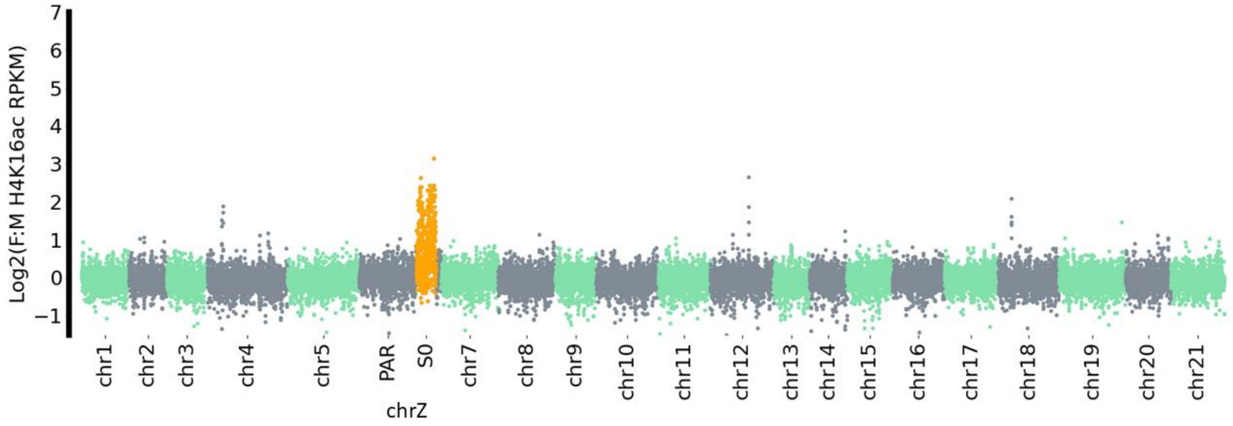

Supplementary Figure 11: Female to male coverage distribution of H4K16ac in 30kb windows across 21 chromosomes in Gonads in *A. franciscana*.

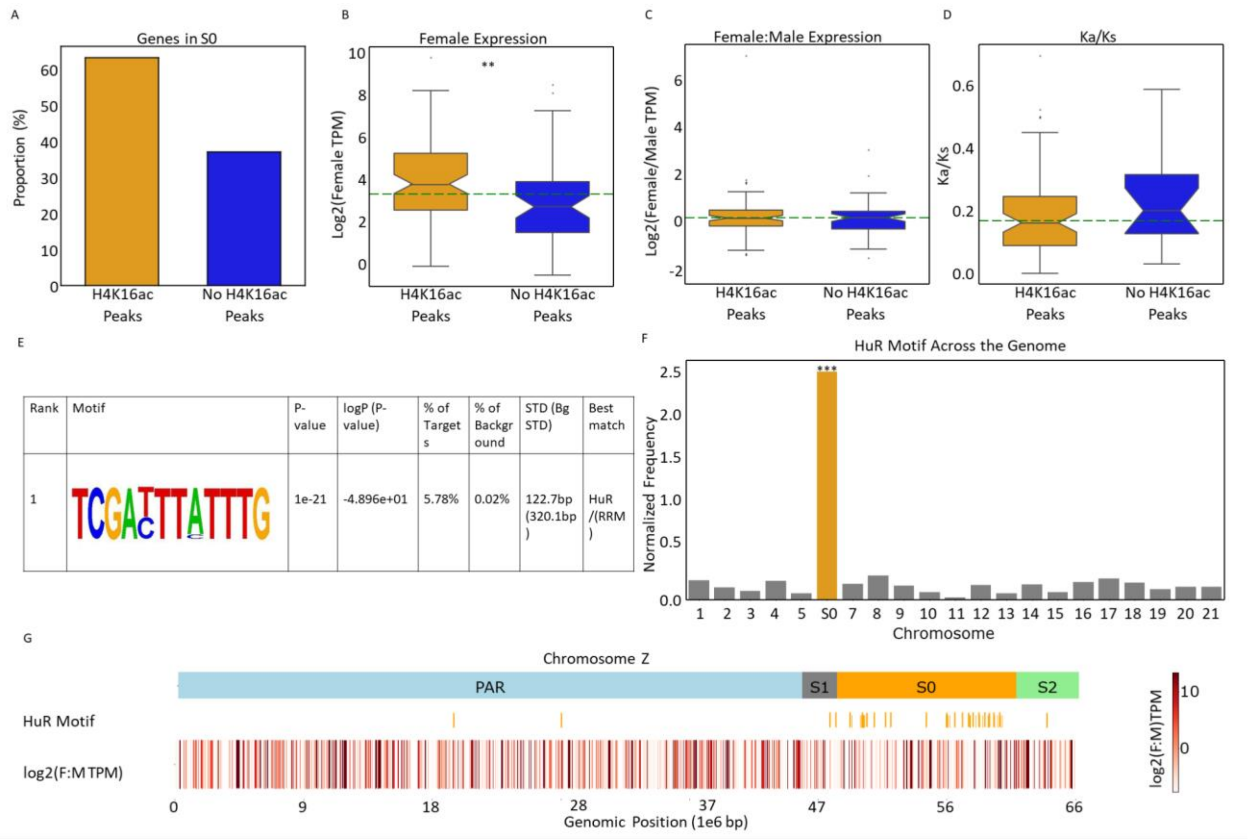

Supplementary Figure 12: **H4K16ac peaks in the S0 region and their association with functional genomic features.** (A) Proportion of genes in the Z-specific region (S0) that either intersect or do not intersect with H4K16ac peaks in female gonads. (B) Expression of genes in the Z-specific region (S0) with and without H4K16ac peaks in female gonads. The significance of Wilcoxon rank sum test ** is *P*-value < 0.005. (C) Log2 of female:male expression ratio of S0 genes with and without H4K16ac peaks in gonads. (D) The distribution of Ka/Ks of S0 genes with and without overlap with H4K16ac peaks. (E) Motif with the most significant enrichment in S0 H4K16ac peaks relative to the rest of the genome. (F) Frequency (in number per million base pairs) of the motif described in (E) on different chromosomes. *** denotes p<0.005, obtained by resampling N chromosomal loci randomly 10000 times, where N is the number of peaks found throughout the genome. (G) Schematic regions of the Z chromosome, HuR motif distribution patterns and their relationship to female-to-male expression ratios in gonadal tissue.

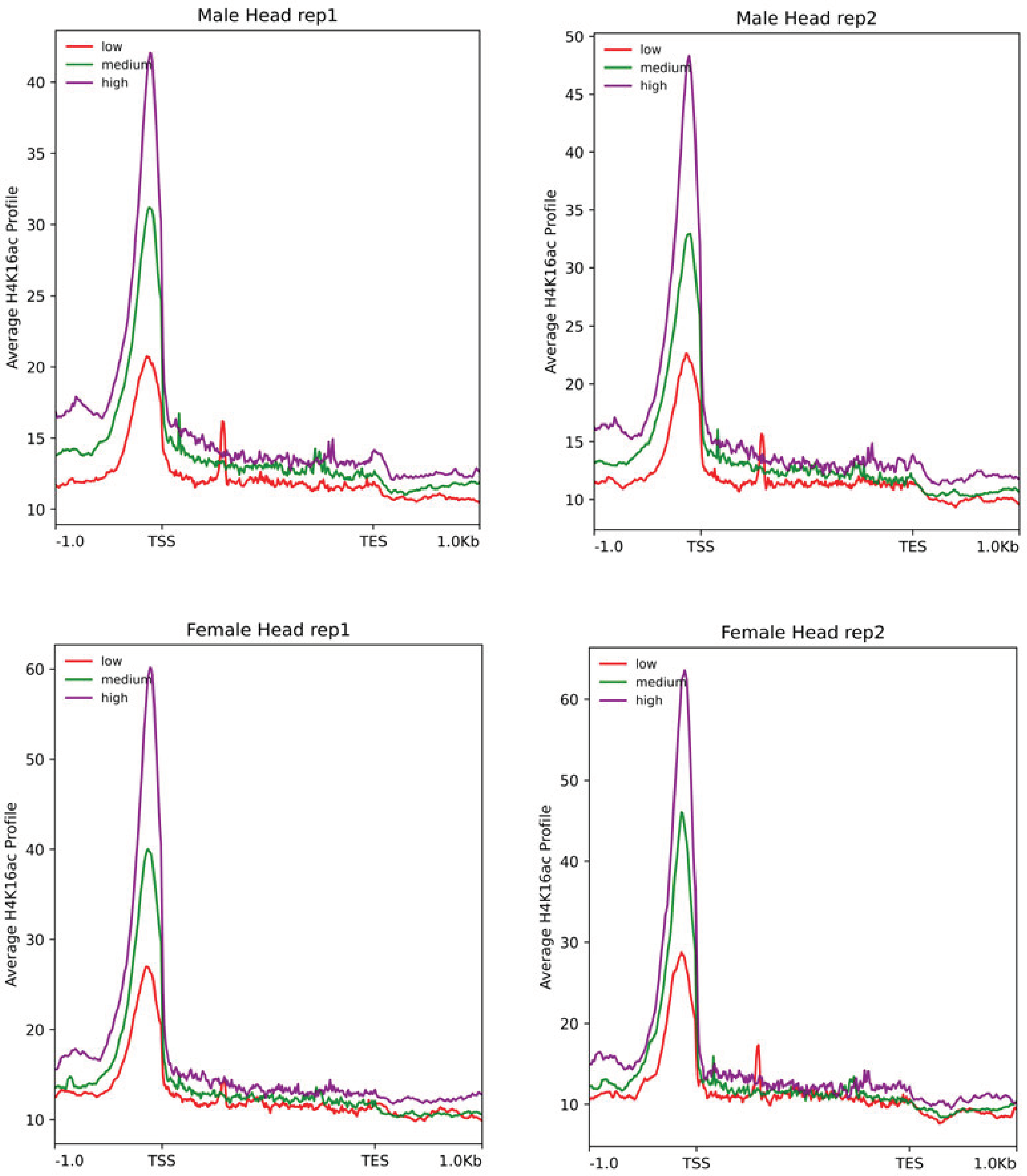
Male Head rep1
Male Head rep2
Female Head rep1
Female Head rep2
low
medium
high
Average H4K16ac Profile
-1.0
TSS
TES
1.0Kb
10
15
20
25
30
35
40
45
50
60

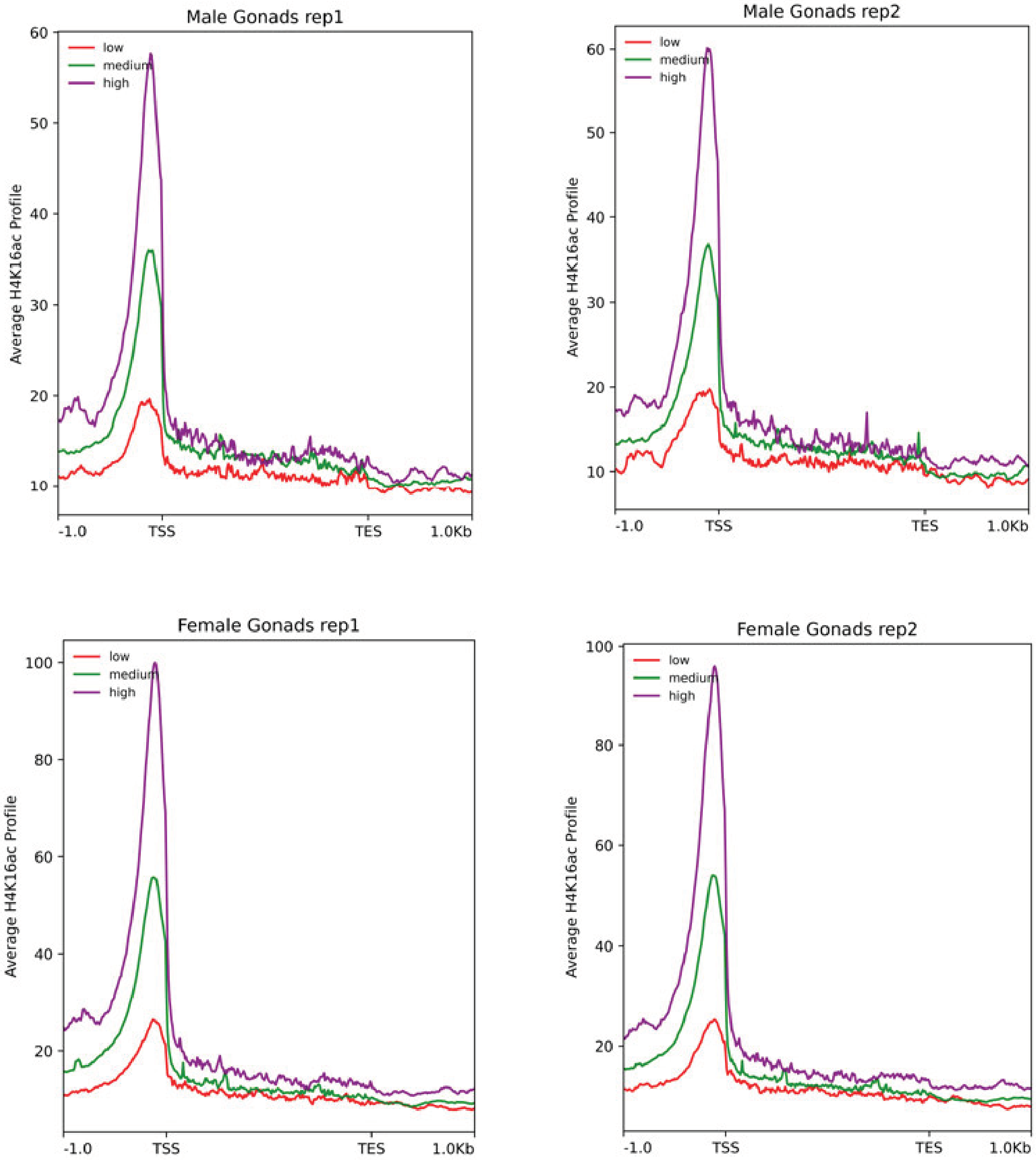

Supplementary Figure 13: Average enrichment of H4K16ac across gene length (only autosomal and PAR genes were considered with TPM > 0.5) of different expression levels (low (those less than 30% in TPM across these genes considered), medium (those genes between 30% and 70%) and high (those above 70%)) in both head and gonads.

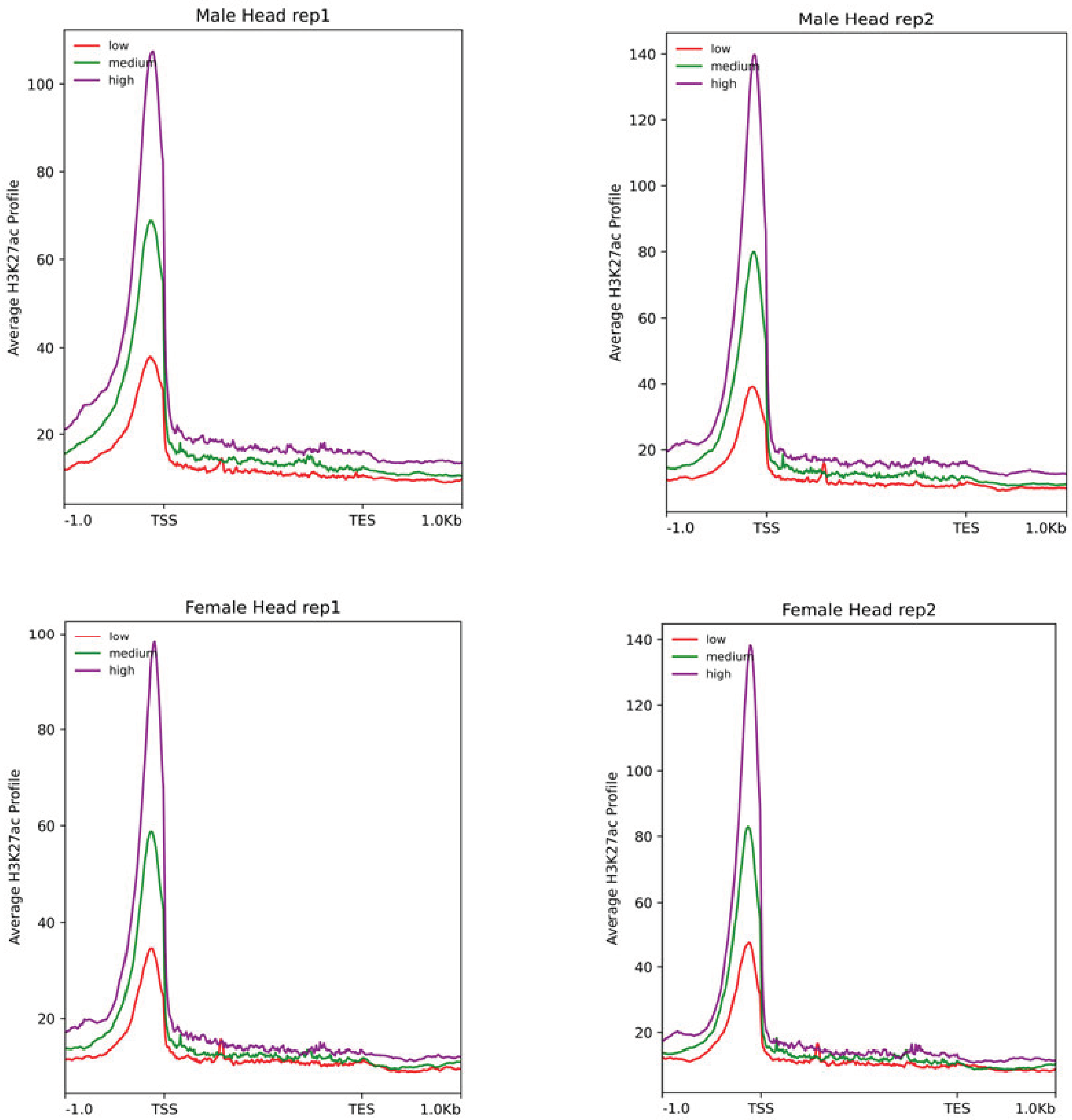

Supplementary Figure 14: Average enrichment of H3K27ac across gene length (only autosomal and PAR genes were considered with TPM > 0.5) of different expression levels (low (those less than 30% in TPM across these genes considered), medium (those genes between 30% and 70%) and high (those above 70%)) in both head and gonads.

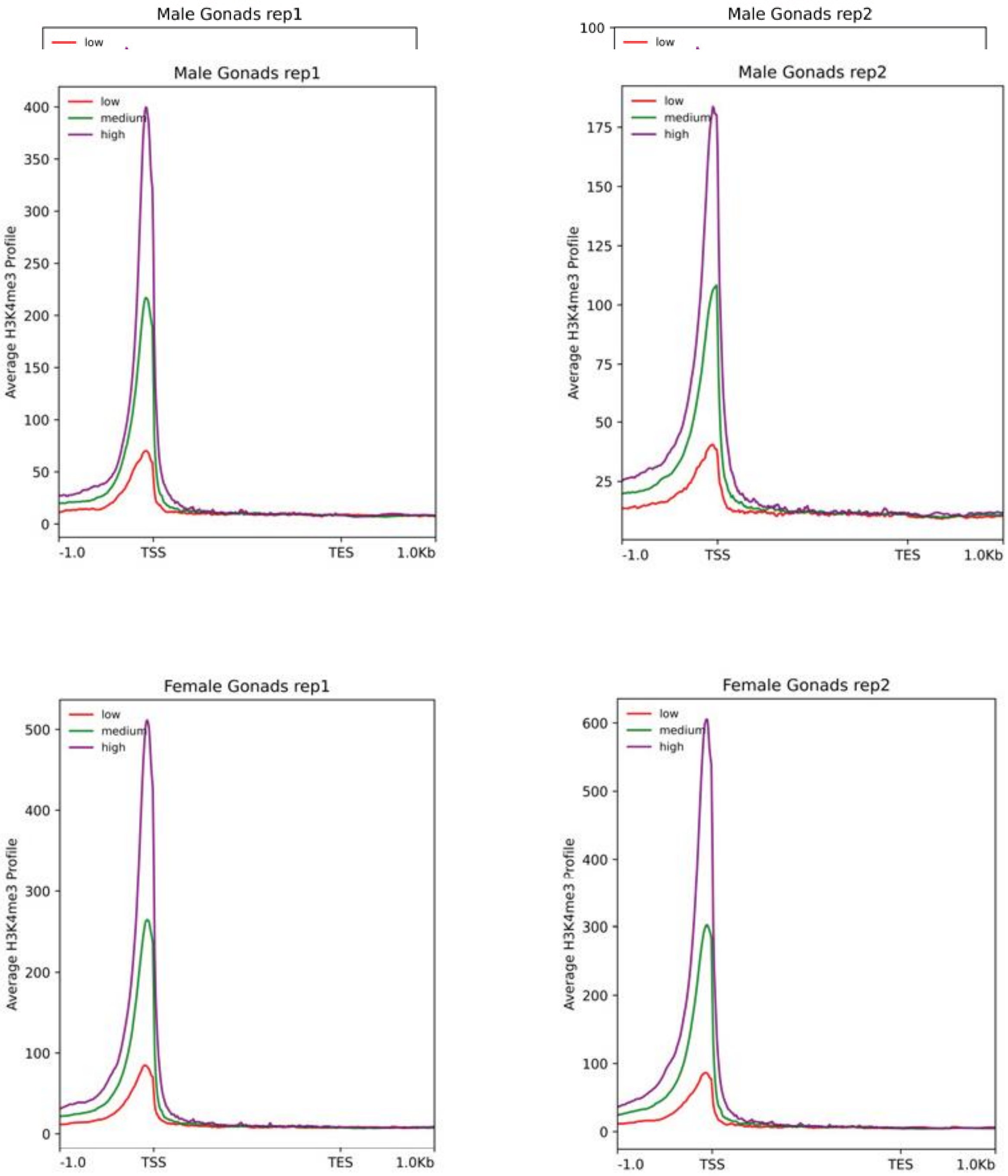
Male Gonads rep1
Male Gonads rep2
low
medium
high
Average H3K4me3 Profile
-1.0
TSS
TES
1.0Kb
Female Gonads rep1
Female Gonads rep2

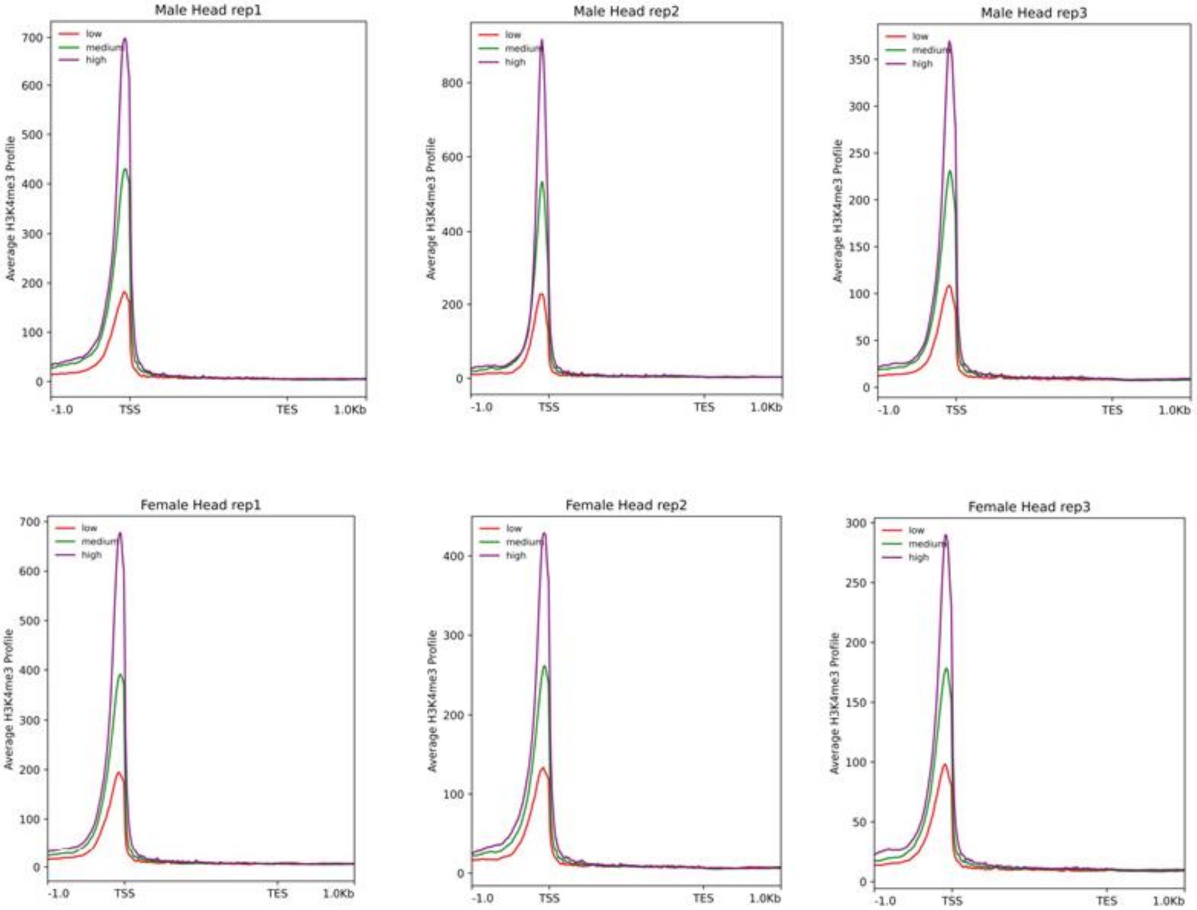

Supplementary Figure 15: Average enrichment of H3K4me3 across gene length (only autosomal and PAR genes were considered with TPM > 0.5) of different expression levels (low (those less than 30% in TPM across these genes considered), medium (those genes between 30% and 70%) and high (those above 70%)) in both head and gonads.

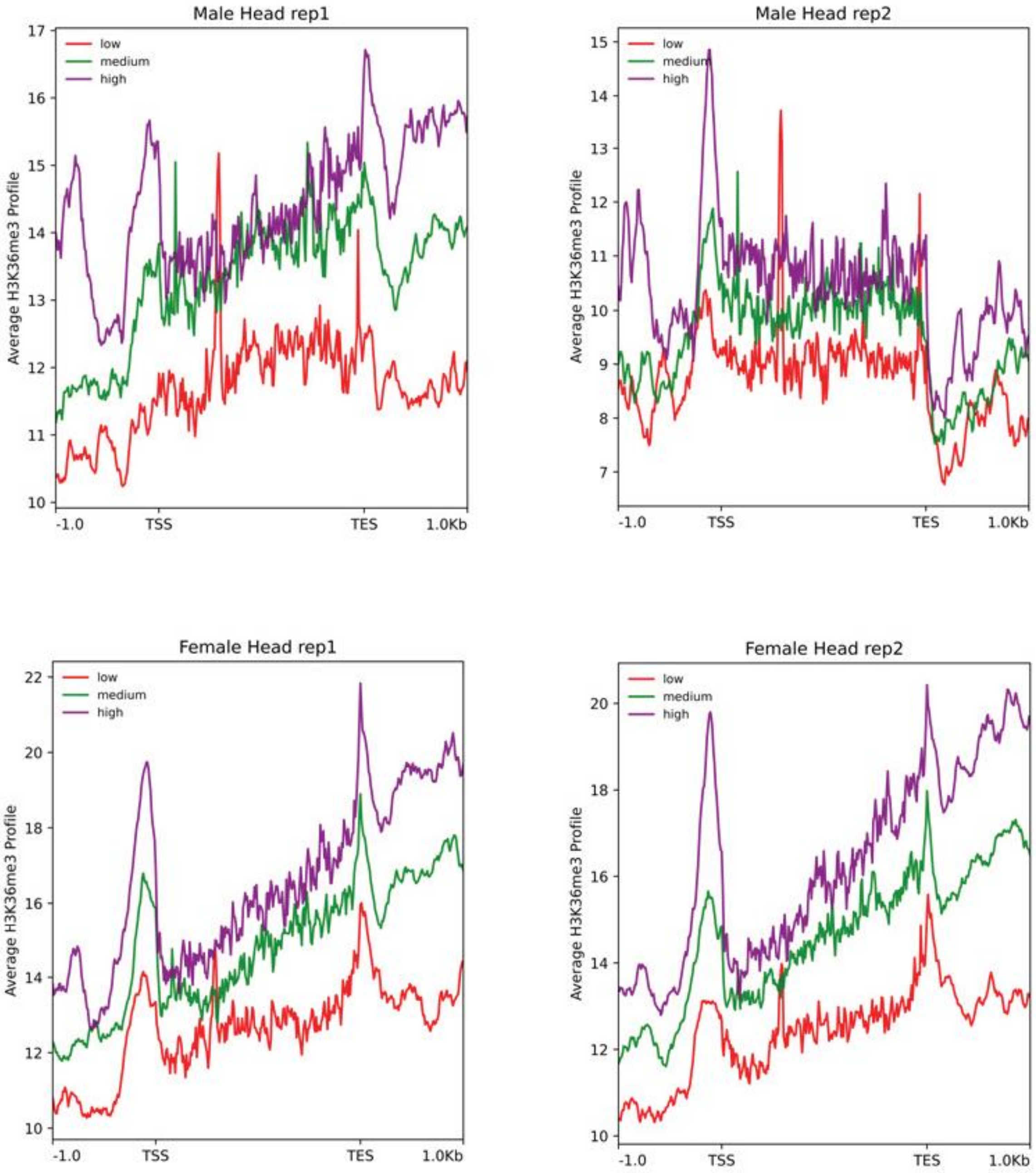
Male Head rep1
low
medium
high
Average H3K36me3 Profile
-1.0
TSS
TES
1.0Kb
Male Head rep2
low
medium
high
Average H3K36me3 Profile
-1.0
TSS
TES
1.0Kb
Female Head rep1
low
medium
high
Average H3K36me3 Profile
-1.0
TSS
TES
1.0Kb
Female Head rep2
low
medium
high
Average H3K36me3 Profile
-1.0
TSS
TES
1.0Kb

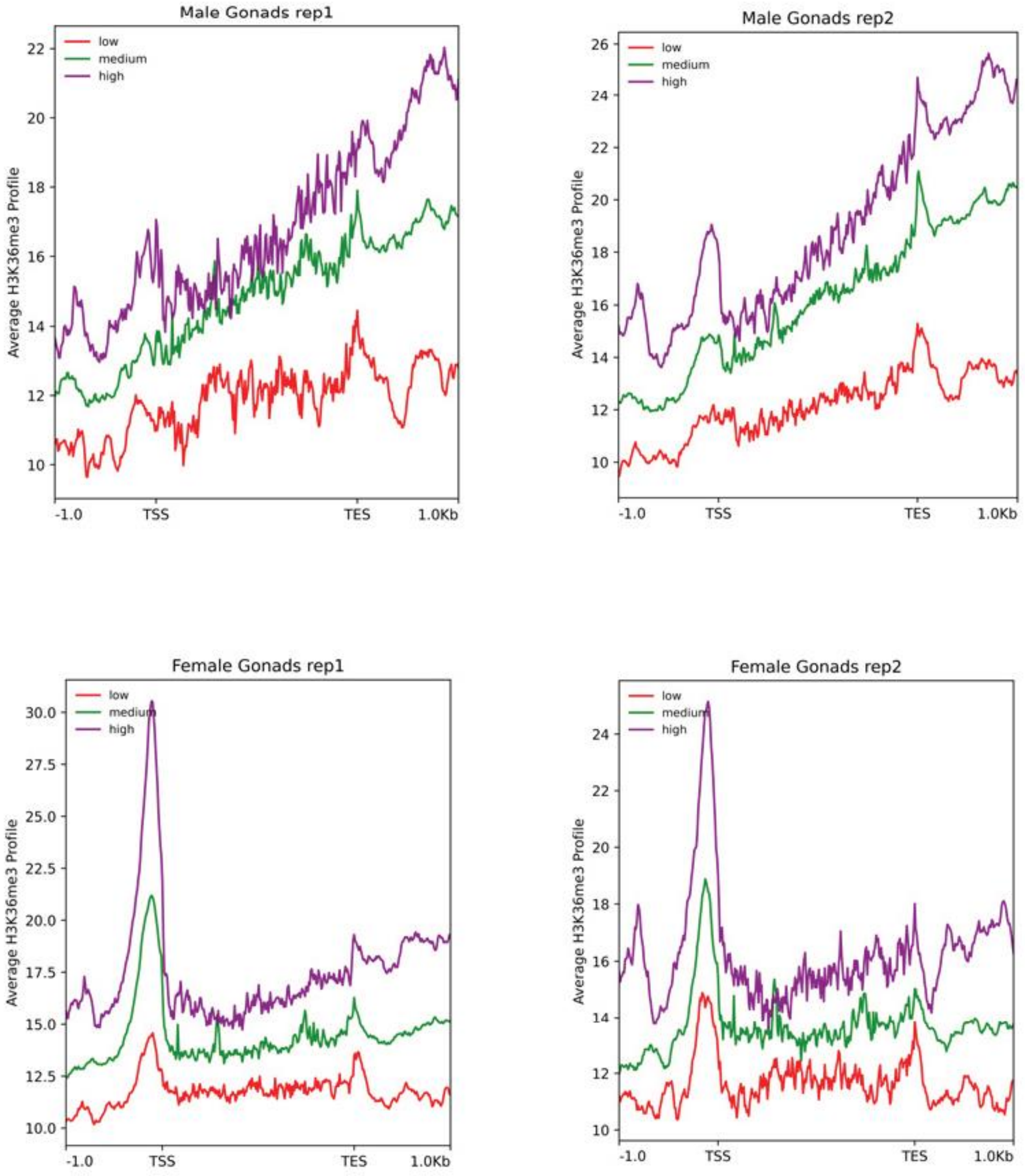

Supplementary Figure 16: Average enrichment of H3K36me3 across gene length (only autosomal and PAR genes were considered with TPM > 0.5) of different expression levels (low (those less than 30% in TPM across these genes considered), medium (those genes between 30% and 70%) and high (those above 70%)) in both head and gonads

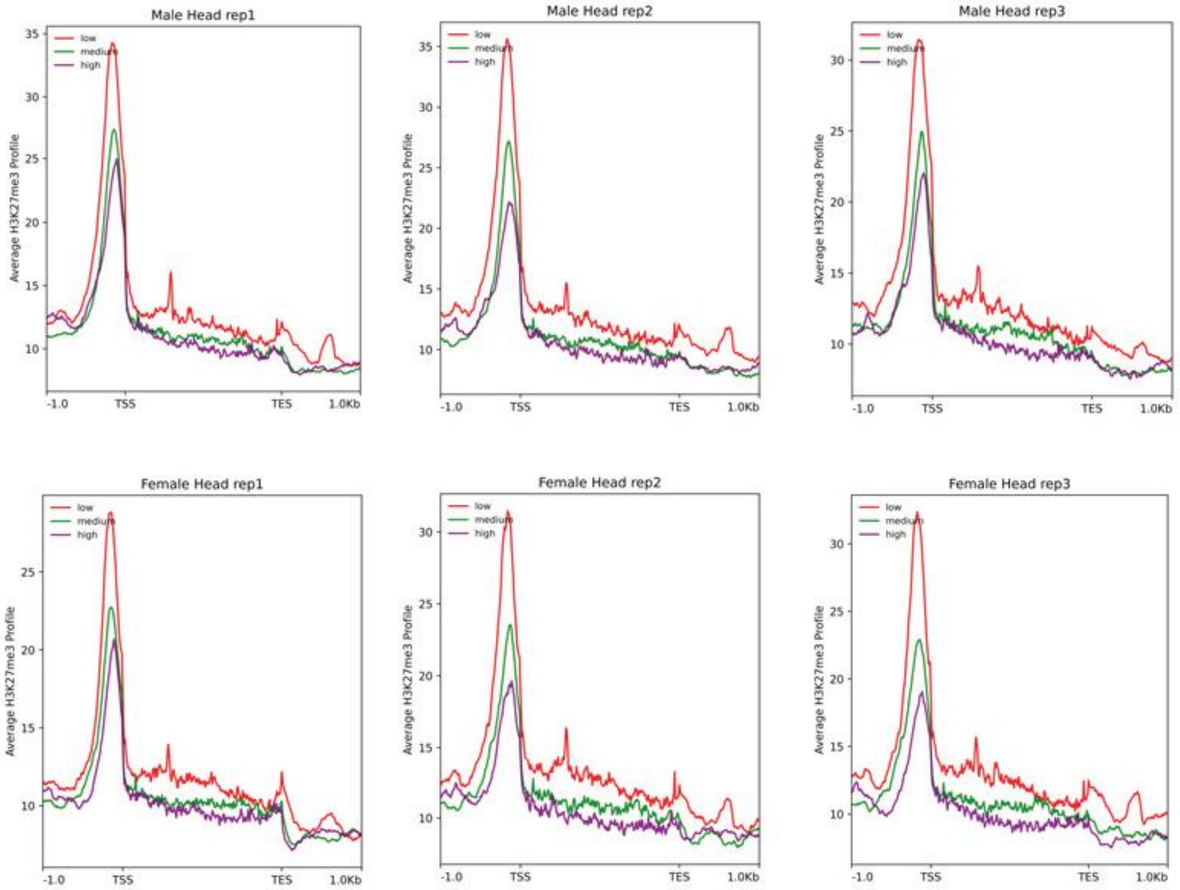
Male Head rep1
Male Head rep2
Male Head rep3
Female Head rep1
Female Head rep2
Female Head rep3
low
medium
high
Average H3K27me3 Profile
-1.0
TSS
TES
1.0Kb

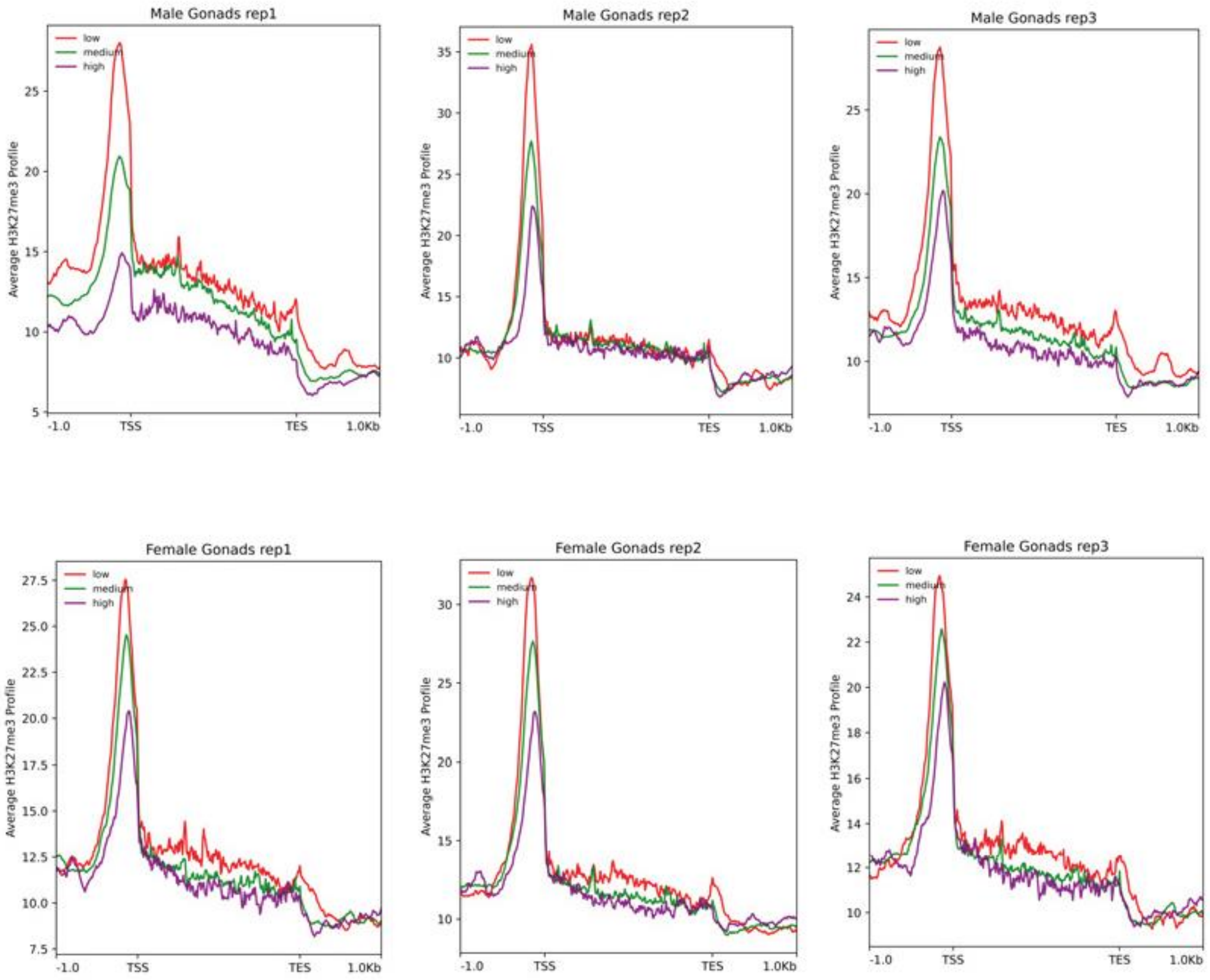

Supplementary Figure 17: Average enrichment of H3K27me3 across gene length (only autosomal and PAR genes were considered with TPM > 0.5) of different expression levels (low (those less than 30% in TPM across these genes considered), medium (those genes between 30% and 70%) and high (those above 70%)) in both head and gonads

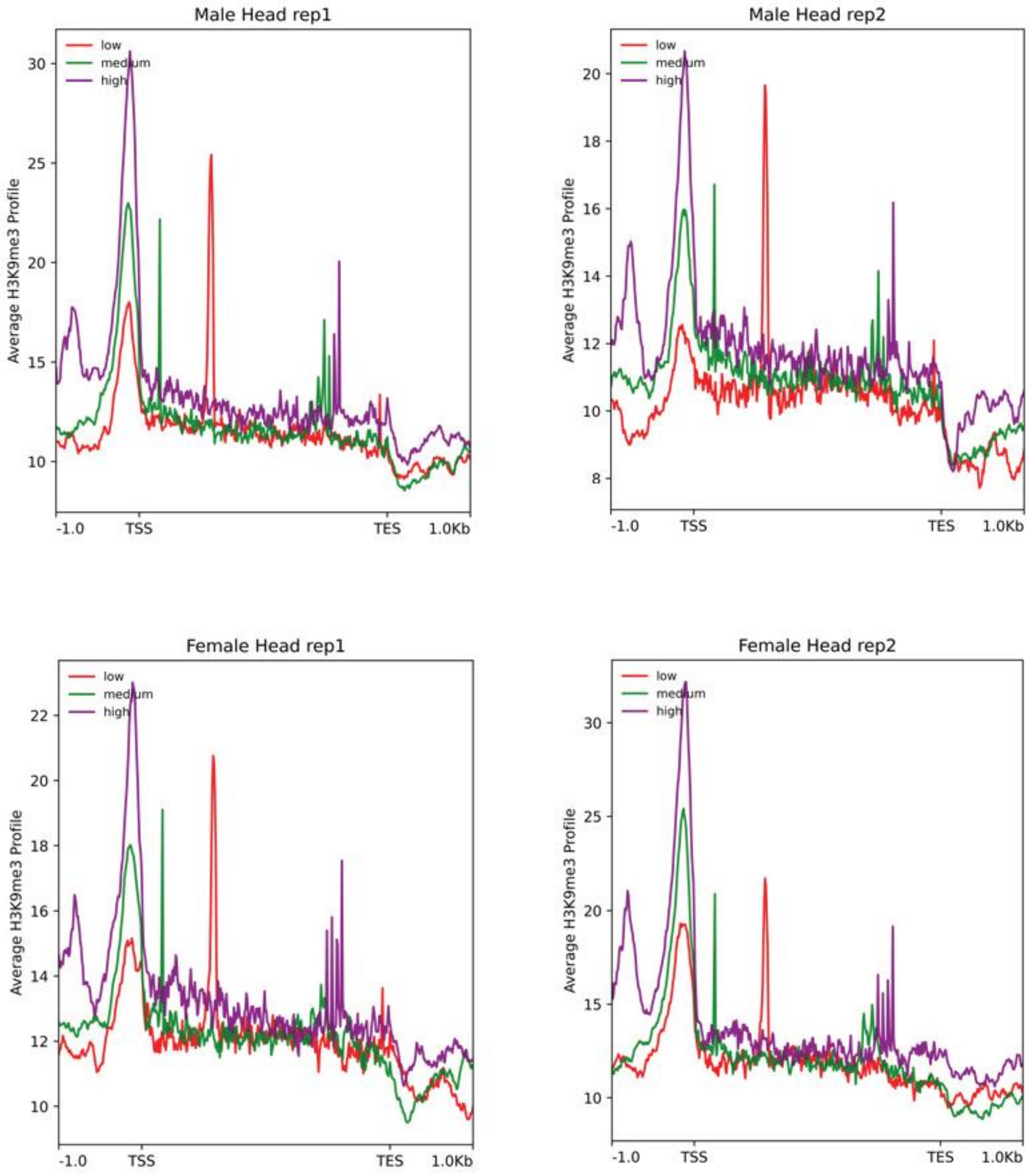
Male Head rep1
Male Head rep2
Female Head rep1
Female Head rep2
low
medium
high
Average H3K9me3 Profile
-1.0
TSS
TES
1.0Kb

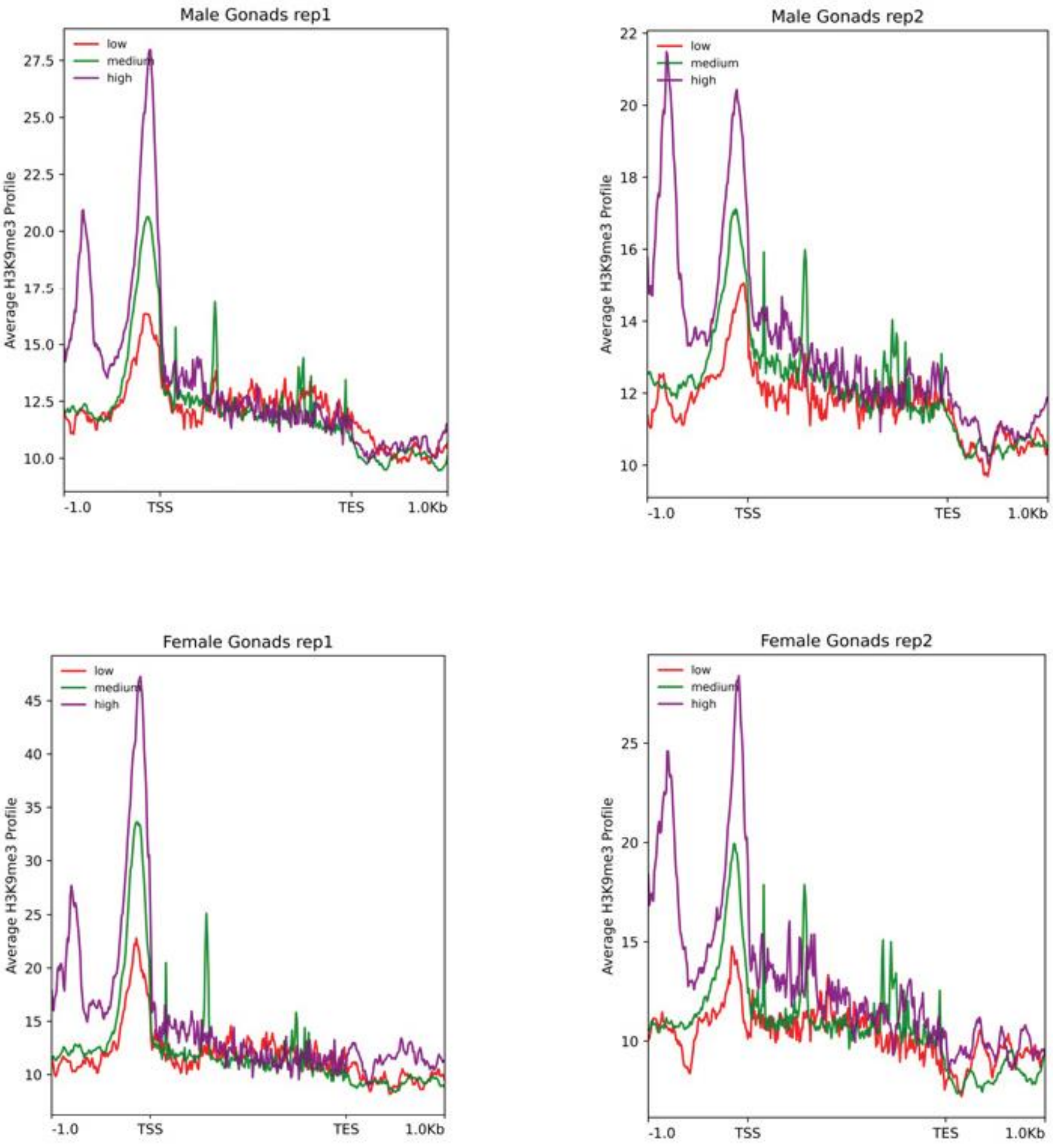

Supplementary Figure 18: Average enrichment of H3K9me3 across gene length (only autosomal and PAR genes were considered with TPM > 0.5) of different expression levels (low (those less than 30% in TPM across these genes considered), medium (those genes between 30% and 70%) and high (those above 70%)) in both head and gonads

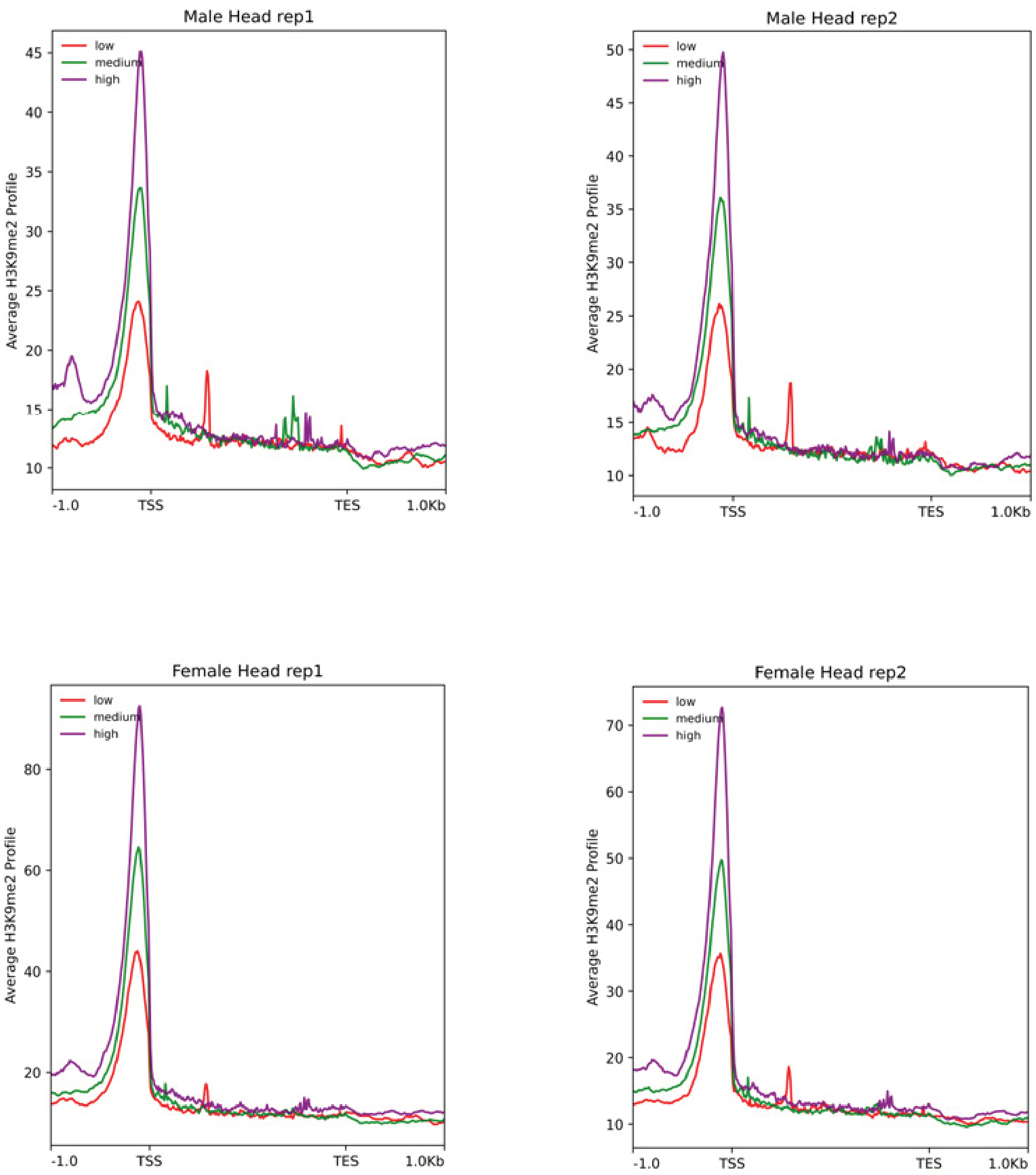
Male Head rep1
Male Head rep2
Female Head rep1
Female Head rep2
low
medium
high
Average H3K9me2 Profile
-1.0
TSS
TES
1.0Kb

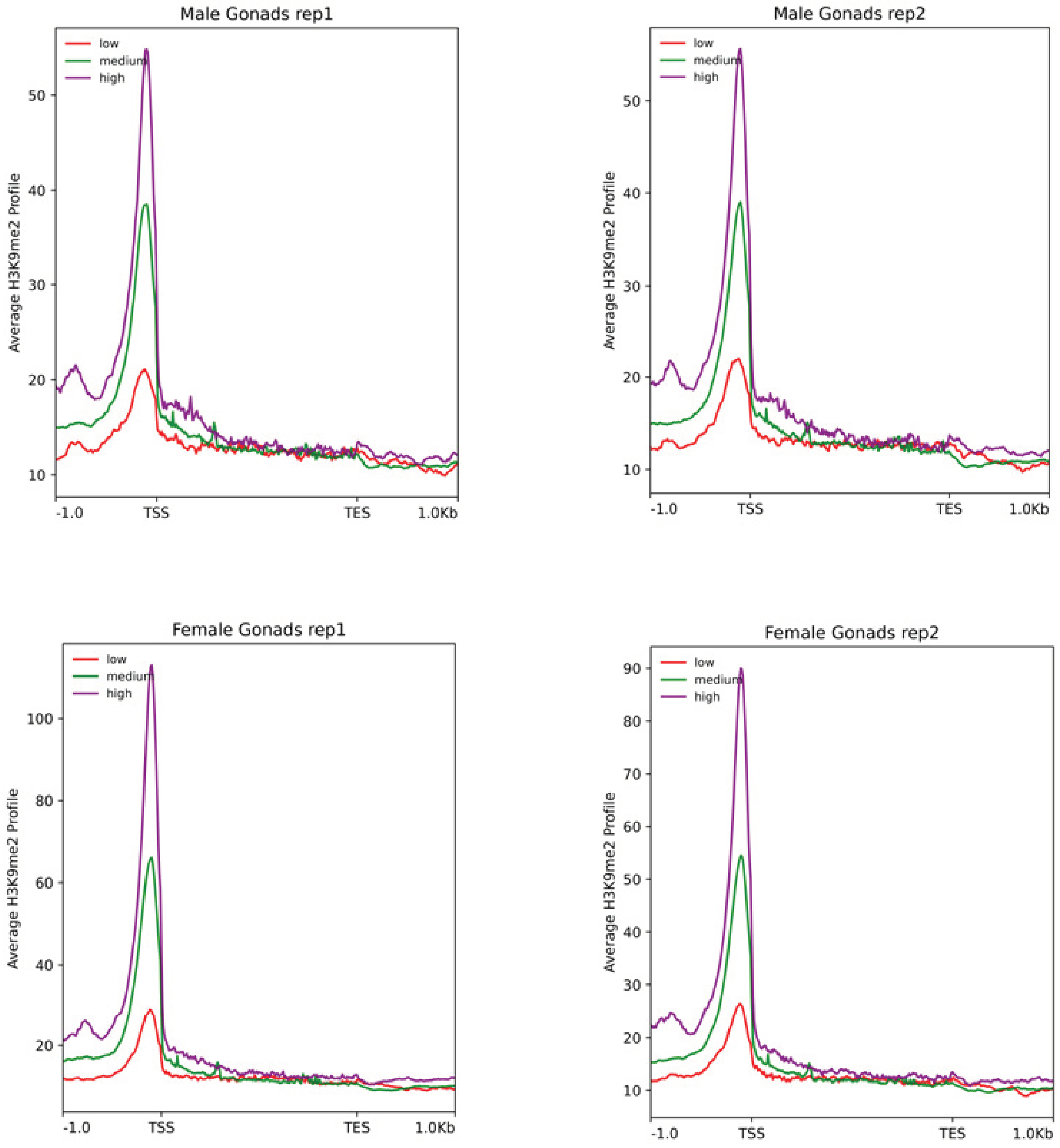

Supplementary Figure 19: Average enrichment of H3K9me2 across gene length (only autosomal and PAR genes were considered with TPM > 0.5) of different expression levels (low (those less than 30% in TPM across these genes considered), medium (those genes between 30% and 70%) and high (those above 70%)) in both head and gonads

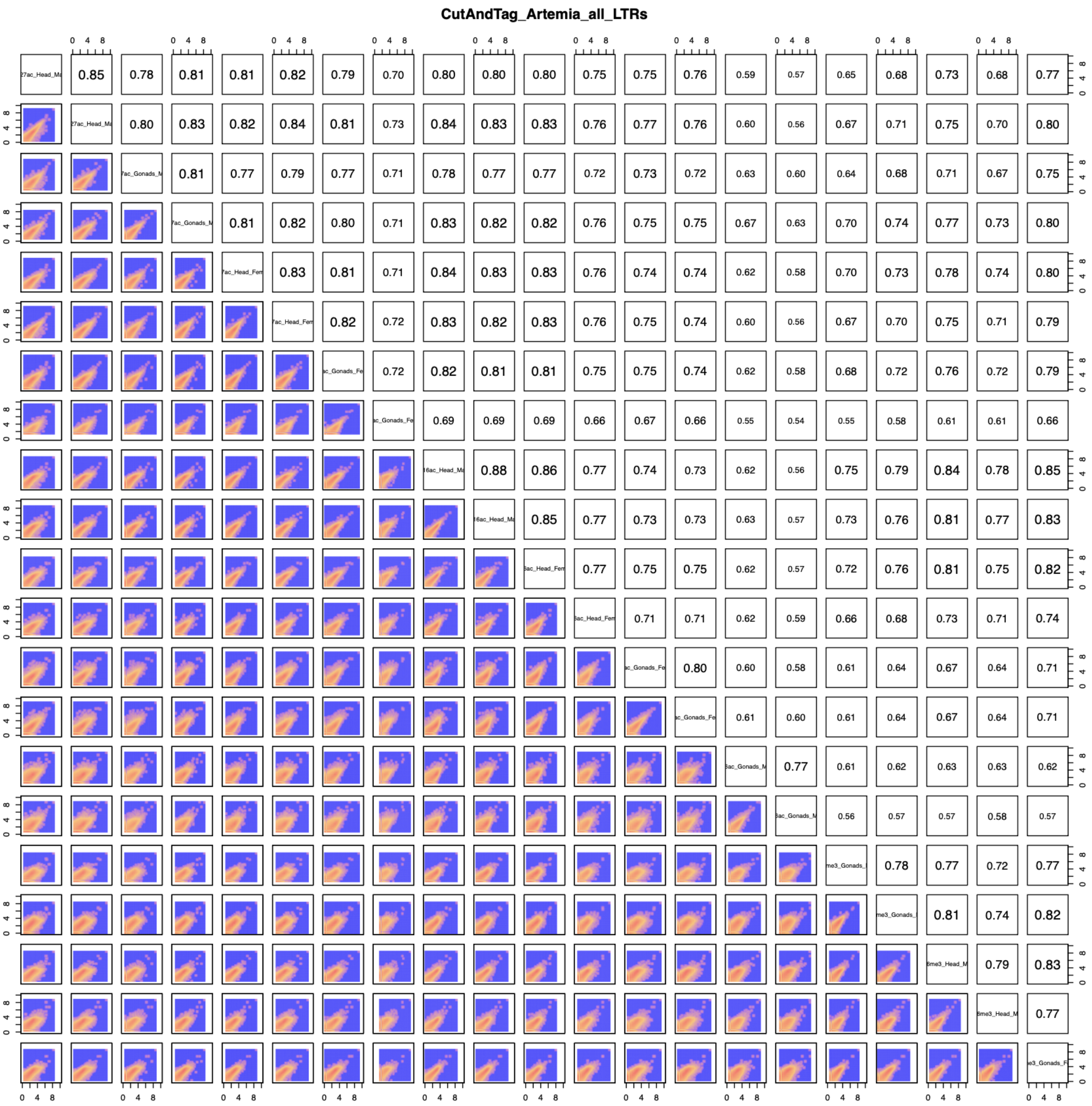

Supplementary Figure 20: Correlation of all samples with all tested histone marks in gonads and heads tissues of both males and females.

Marks	Tissue	Sex	TEs introns	r-value	z-value	z-observed	*P*-value of z-diff
H3K9me3	Heads	Male	Yes<br>No	0.200<br>-0.02	0.202<br>-0.02	14.87	4.89e-50
		Female	Yes<br>No	0.15<br>-0.03	0.151<br>-0.03	12.1	1.1e-33
	Gonads	Male	Yes<br>No	0.16<br>-0.04	0.141<br>-0.04	12.08	1.29e-33
		Female	Yes<br>No	0.14<br>-0.14	0.1409<br>-0.141	18.82	5.07e-79
H3K9me2	Heads	Male	Yes<br>No	0.18<br>-0.03	0.182<br>-0.03	14.15	1.71e-45
		Female	Yes<br>No	0.20<br>0.03	0.203<br>0.03	11.53	8.9e-31
	Gonads	Male	Yes<br>No	0.20<br>-0.02	0.2027<br>-0.02	14.87	4.8e-96
		Female	Yes<br>No	0.22<br>0.05	0.223<br>0.05	11.593	4.45e-31

Supplementary Table 1: r-z transformations of coefficients of H3K9me3 and H3K9me2 enrichment and expression those genes with TEs in introns and those without TEs in introns oin heads and gonads

Type	Tissue	Autosomes	Pseudo-autosomal (PAR)	Z-linked regions	W-linked genes
Male-biased	Gonads	926	31	22	0
Female-biased	Gonads	594	26	6	7
Male-biased	Heads	10	1	0	0

Female-biased	Heads	10	0	0	5

Supplementary Table 2: sex-biased gene expression in heads and gonads of *A. franciscana* (FDR <0.05, Foldchange >2 & TPM >0.1 for genes in *A. franciscana*)

https://github.com/vkb25/Chromatin-landscape-in-Artemia-franciscana/blob/c8aff7a95879177f6451a347f3dd1c52eee1e2cc/female_biasedchromatintranscriptrep_transcript.xlsx

Supplementary Table 3: GO terms of contrasting chromatin states and female-biased gene expression in gonads of *A. franciscana*

https://github.com/vkb25/Chromatin-landscape-in-Artemia-franciscana/blob/c8aff7a95879177f6451a347f3dd1c52eee1e2cc/male_biasedchromatintranscriptrep_transcript.xlsx

Supplementary Table 4: GO terms of contrasting chromatin states and male-biased gene expression in gonads of *A. franciscana*

https://github.com/vkb25/Chromatin-landscape-in-Artemia-franciscana/blob/f31ed9a097cfeec0c37a5fd27d47cd8de3808388/Gonads_homerResults.html

Supplementary Table 5: Transcription factor binding sites with significant enrichment within H4K16ac CUT&TAG peaks in female gonadal tissues. Analysis was performed using HOMER

https://github.com/vkb25/Chromatin-landscape-in-Artemia-franciscana/blob/f31ed9a097cfeec0c37a5fd27d47cd8de3808388/Somatic_homerResults.html

Supplementary Table 6: Transcription factor binding sites with significant enrichment within H4K16ac CUT&TAG peaks in female head tissues. Analysis was performed using HOMER

https://github.com/vkb25/Chromatin-landscape-in-Artemia-franciscana/blob/d9e9091814fe0611e2ccab2f67a752a25f596f53/supplementary%20files%20on%20mapping%20reads.xlsx

Supplementary Table 7: Total reads mapped, alignment rate, estimated library size and the amount of mapped reads retained for downstream analysis after applying strict filtering steps

Sample ID	Sex	Tissue
SRR8641214	Female	Ovaries
SRR8641213	Female	Ovaries
SRR8641212	Female	Head
SRR8641211	Female	Head
SRR8641218	Male	Testes
SRR8641217	Male	Testes
SRR8641216	Male	Head
SRR8641215	Male	Head

Supplementary Table 8: RNA raw reads together with their SRA numbers used for expression analysis.
